# Challenges of Studying Amelogenesis in Gene-Targeted Mouse Models

**DOI:** 10.3390/ijms26104905

**Published:** 2025-05-20

**Authors:** Charles E. Smith, John D. Bartlett, James P. Simmer, Jan C.-C. Hu

**Affiliations:** 1 Department of Anatomy & Cell Biology, Faculty of Medicine & Health Sciences, McGill University, 3640 University St., Montreal, QC H3A 0C7, Canada; charles.smith@mcgill.ca; 2 Department of Biologic and Materials Sciences, University of Michigan School of Dentistry, 1011 North University Ave., Ann Arbor, MI 48190, USA; jsimmer@umich.edu; 3 Division of Biosciences, College of Dentistry, Ohio State University, 305 W. 12th Ave., Columbus, OH 43210, USA; bartjd20@gmail.com

**Keywords:** enamel formation, amelogenesis imperfecta, basement membrane, genetically modified mouse models, enamel organ, ameloblasts

## Abstract

Research on how a stratified oral epithelium gained the capability to create the hardest hydroxyapatite-based mineralized tissue produced biologically to protect the surfaces of teeth has been ongoing for at least 175 years. Many advances have been made in unraveling some of the key factors that allowed the innermost undifferentiated epithelial cells sitting on a skin-type basement membrane to transform into highly polarized cells capable of forming and controlling the mineralization of the extracellular organic matrix that becomes enamel. Genetic manipulation of mice has proven to be a useful approach for studying specific events in the amelogenesis developmental sequence but there have been pitfalls in interpreting loss of function data caused in part by conflicting literature, technical problems in tissue preservation, and the total amount of time spent on tooth development between different species that have led to equivocal conclusions. This critical review attempts to discuss some of these issues and highlight the challenges of characterizing amelogenesis in gene-targeted mouse models.

## 1. Introduction

### 1.1. Scope of This Review

Enamel formation is viewed by many cellular and molecular biologists as organ-specific and at the outer fringe of mineralized tissue research. This is mostly due to unfamiliarity with enamel, how it is formed, and the complex series of sequential steps that a specialized group of epithelial cells—the ameloblasts—must pass through in order to produce it. Presently, a commonly used approach available to investigate the mechanisms of how ameloblasts make enamel is by mutating or silencing genes in mice to see what happens. This paper is not about the mechanisms of enamel formation per se, but it is about the tool being used to investigate these mechanisms. Mistakes have been made because researchers do not always take into account fine details about numerous cellular changes progressively occurring as enamel develops and the fact that poor fixation of genetically altered mice can be misinterpreted as indicative of an enamel defect. Although in one sense enamel is enamel, irrespective of what tooth it serves as a protective covering for, researchers can easily distinguish molar enamel from incisor enamel in mice just by its microscopic structural appearance. There are clearly many other factors at play, which is the focus of this review. Our objective is to discuss the advantages and limitations of characterizing amelogenesis to define enamel defects of gene-targeted mouse models.

**Figure 1:**
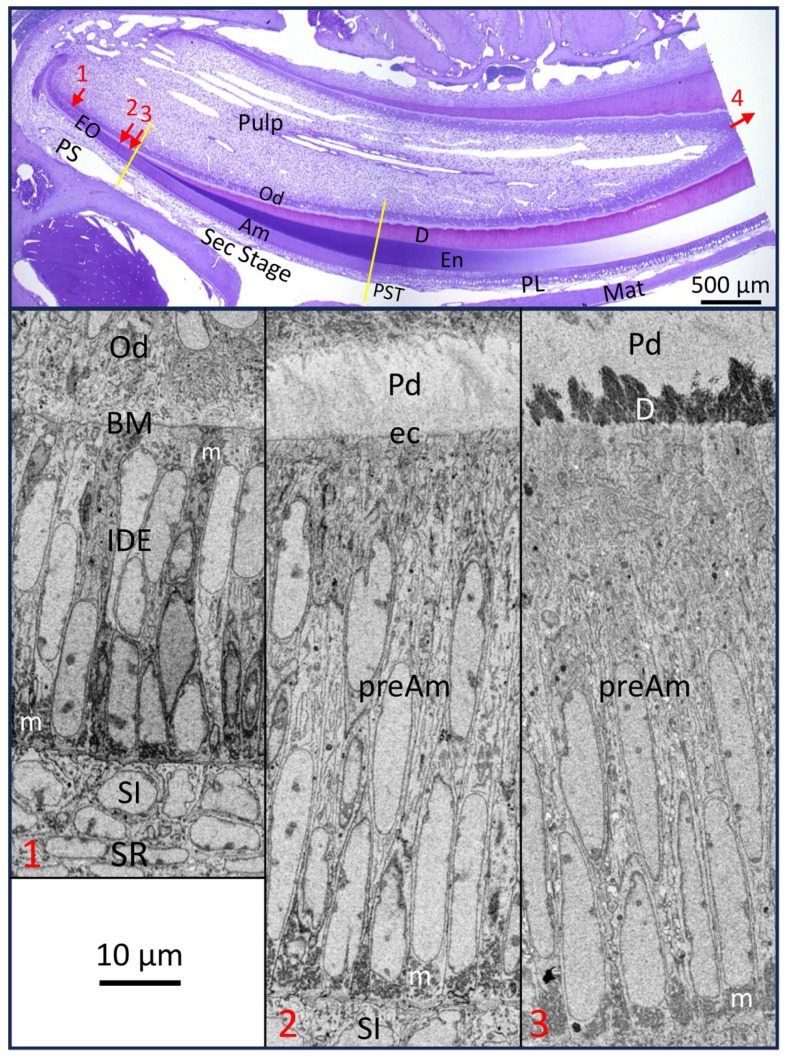
Mandibular mouse incisor showing major reference points in amelogenesis. (**Top**) 0.5 µm thick plastic longitudinal section of a decalcified tooth stained with toluidine blue showing roughly 75% (6 to 8 mm) of the part of the continuously renewing incisor embedded in alveolar bone. (**Bottom**) show inverted backscatter FIB-SEM images of portions of the enamel organ (EO) at 3 locations (numbers in red correspond with arrow numbers in the top panel across the length of presecretory stage (PS), where inner dental epithelium (IDE) cells progressively transform into taller preameloblasts (preAm)). *Arrow 1*: IDE cells face differentiating odontoblasts (Obs) across a skin-type basement membrane (BM); *Arrow 2*: site where finger-like projections from preAm burrow through the BM to remove it by endocytosis (ec) (see Figure 2D); *Arrow 3*: shortly thereafter, mineral is seeded in the predentin (Pd) to create a continuous mineral layer and a mineralization front for dentin (D). *Arrow 4* shows the direction of incisor eruption. Ameloblasts start forming the enamel layer by appositional growth during the secretory stage (Sec) and thereafter by assisting the massive growth in the volume of millions of hydroxyapatite crystallites seeded during the Sec stage after undergoing postsecretory transition (PST) into the maturation stage (Mat) (**Top**). Here, the distal ends of ameloblasts rhythmically modulate between ruffle-ended (the predominate state) and smooth-ended morphologies needed in part to keep the pH within the enamel layer from becoming too acidic and in part to flush out fragments of former enamel proteins being cleaved by KLK4 protease secreted by the modulating ameloblasts (En: enamel layer; PL: papillary layer of EO during Mat stage; m: mitochondria). This figure includes images first published in Smith et al., 2016 [1].

**Figure 2:**
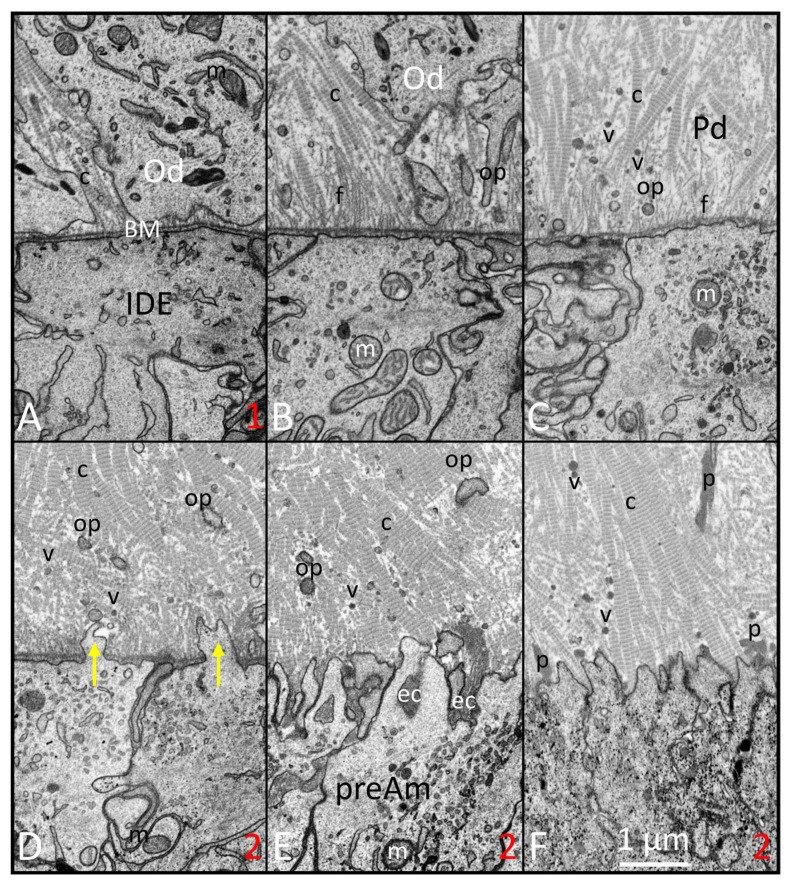
FIB-SEM images of the presecretory stage illustrating the time forward sequence of changes (**A**–**F**) happening along the interface between inner dental epithelium (IDE) and odontoblasts (Ods). (**A**) (similar to site 1 in Figure 1): IDE cells appear primitive in organelle content. The lamina densa of the basement membrane (BM) is uniform and smooth just outside the distal surface of the IDE cells. The differentiating odontoblasts (Ods) have numerous small collagen fibrils (c) intercellularly positioned between neighboring Ods. (**B**) Ods move away from the BM inwards toward the pulp and leave behind the beginnings of the odontoblastic processes (ops). The newly forming predentin contains numerous delicate unbanded fibrils (fs) extending between the lamina densa and thin collagen (c) fibrils. (**C**) Numerous matrix vesicles (v) and small odontoblastic processes are seen in the thickening predentin (Pd) layer. There appear to be fewer fine filaments (fs) and transformation of collagen (c) fibrils into thicker banded fibers. (**D**–**F**) (similar progressively forward locations can be seen at site 2 in Figure 1): small finger-like protrusions start to project through the BM (yellow arrows) in the areas formerly occupied by the fine fibrils (**D**). Remnants of the BM then are endocytosed (ec) into preameloblasts (preAm; (**E**)). Once cleared, the enlarging collagen fibers come into intimate contact with the undulating distal membrane of the preAm. Pools of protein (p) of various sizes, mostly comprising amelogenins, start to accumulate on the predentin (Pd) side of the preameloblasts as well as deeper within the Pd (**F**) (m: mitochondria). This figure, containing images with improved resolution, was first published in Smith et al., 2016 [1].

**Figure 3:**
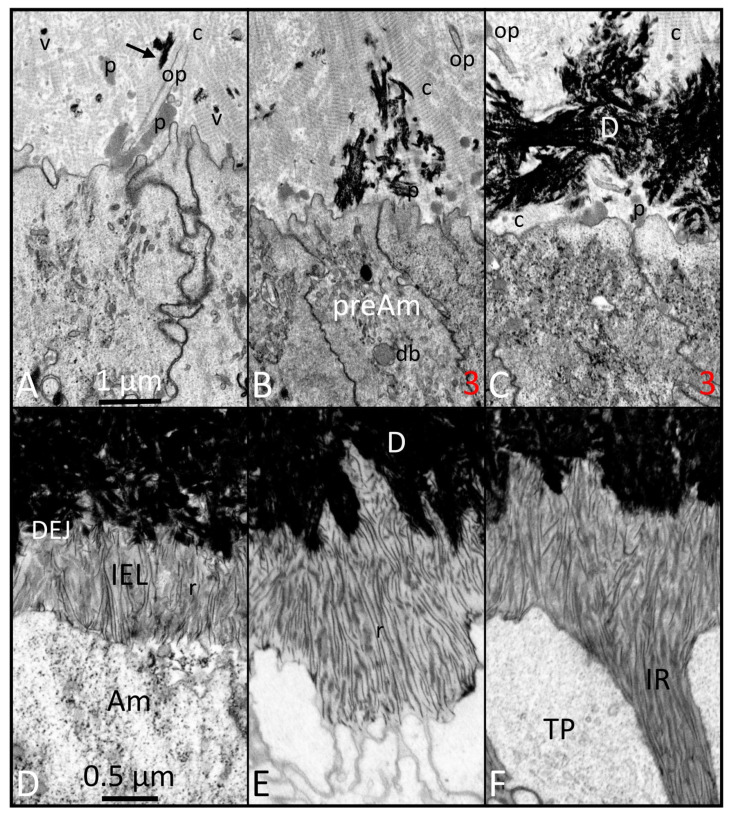
FIB-SEM images of the start of dentin mineralization late in the presecretory stage ((**A**–**C**); same magnification): early phases of appositional growth of the enamel layer in the secretory stage ((**D**–**F**); same magnification). (**A**) Initial mineral deposits in the predentin occur as small spherical clusters inside matrix vesicles (vs) or as elongated linear rods (black arrow) between or on the surface of collagen fibers (c). No mineral forms in relation to the protein clusters (p). (**B**,**C**) (similar to site 3 in Figure 1): Mineralization spreads rapidly both laterally and vertically. Some areas of the predentin closest to the distal surface of preameloblasts remain mineral-free for a slightly longer time than neighboring areas (db: dense body [lysosome]). (**D**) As ameloblasts (Ams) move outward away from the DEJ, they leave behind thin mineral ribbons (rs) that appear to be attached at one end to the mineral in dentin. These ribbons are arranged mostly vertically and parallel to each other, and form part of a thin innermost layer called the initial enamel layer (IEL). (**E**) A differential rate of ribbon lengthening starts to occur at sites down the sides between adjacent Ams, which encases the distal end of the ameloblast to map out a structure called the Tomes process (TP), a cytoplasmic structure responsible for forming the enamel rods. (**F**) The lateral extensions of mineral ribbons from the IEL are called enamel prongs and form territories of enamel mineral that are called interrod (IR) enamel. This figure, containing images with improved resolution, was first published in Smith et al., 2016 [1].

**Figure 4:**
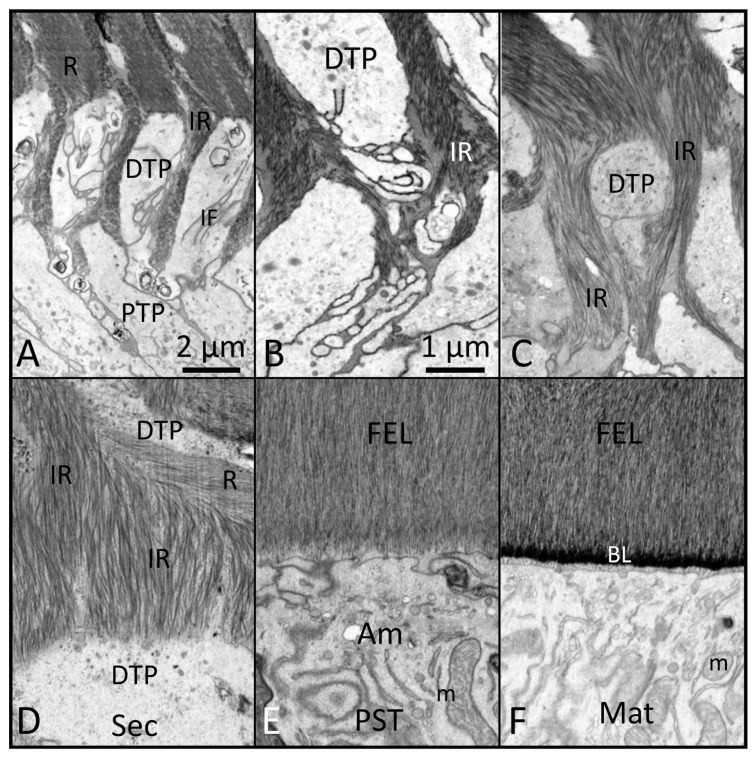
FIB-SEM images of mid (**A**–**C**) to late (**D**) secretory-stage enamel formation (Sec), followed by postsecretory transition (PST) (**E**) into early maturation (Mat) (**F**) (magnification bar in Panel (**B**) applies to (**B**–**F**)). (**A**) Once the Tomes processes become functional, they can be divided into a proximal part (PTP) and a distal part (DTP). The DTP projects into the developing enamel layer and shows many infoldings (IF) of the outer plasma membrane. (**B**,**C**) As formation of the inner enamel layer proceeds, the arrangement of mineral crystallites within the interrod (IR) becomes very complex, including many bends and curvatures. (**D**) IR forms a greater portion of enamel volume during the formation of the outer enamel layer and includes many bends and curvatures to the constituent crystallites. (**E**) Towards the end of the secretory stage, ameloblasts (AMs) lose their Tomes processes and create an outer final enamel layer (FEL). Like within the initial enamel layer (Figure 3D), the crystallites of the FEL tend to run vertically and parallel and terminate in close proximity to the distal membranes of AMs as they undergo postsecretory transition (PST). (**F**) Near the end of PST, the Ams put back onto the enamel surface a sugar-rich basal lamina (BL) that appears to be blended into the mineralized area as opposed to sitting on its surface. The BL is carried forward and remains in position throughout the maturation stage (m: mitochondria). This figure, containing images with improved resolution, was first published in Smith et al., 2016 [1].

**Figure 5:**
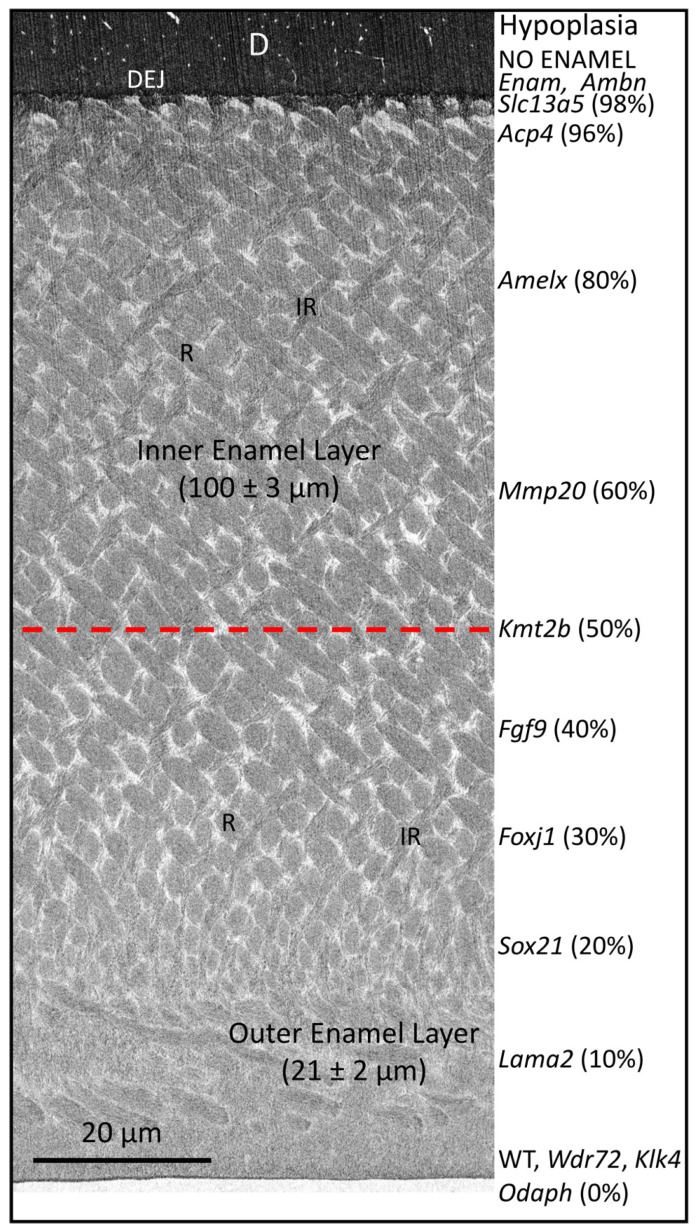
FIB-SEM image of early maturing enamel from a mandibular mouse incisor showing its full thickness and proportion as inner enamel, where rods decussate (cross over) in alternating angles, compared with outer enamel, where rods are parallel to each other and angled toward the surface. On the right-hand side of the figure is a list of genes associated with varying levels of hypoplasia when they are dysfunctional (see Table 1). This figure is intended for illustrative purposes only and does not intend to imply that normal enamel structure is simply “shaved off” at the level indicated. In reality, varying amounts of rod/interrod disorganization occur within the enamel layer, in most cases in direct proportion to the level of hypoplasia. For example, the mandibular incisors of mice with defective *Slc13a5* genes have among the most severe levels of hypoplasia (98% of the enamel layer missing). The mineralized covering on top of the dentin is not true enamel but something less mineralized, more like acellular cementum than true enamel. In cases where the enamel organ becomes very dysplastic as part of the problem, the mineralized material on dentin is more analogous to an ectopic (random) mineralized crust. In contrast, mandibular incisors of mice with defective *Klk4* or *Odaph* genes have an enamel layer of full thickness and with rod (R) and interrod (IR) patterns exactly as illustrated in this figure. The problem in this case is hypomineralization across the maturation stage, resulting in the eruption of teeth covered with soft and easily abraded enamel. This figure, originally published in Bartlett et al., 2021 [2], has been modified.

While preparing this review, we consulted over 350 papers published in PubMed from 1985 to the present, using search terms such as amelogenesis, enamel formation, gene-targeted mouse models, and characterization of enamel defects, between July and December 2024. We critically assessed and incorporated observations from 100 seminal papers, focusing on the ameloblast lifecycle, understanding mutational effects, evaluating ameloblast responses, and drawing conclusions. The 100 papers cited in this review provided foundational knowledge, including insights into cellular and structural development, stage-specific disturbance of ameloblasts, and enamel structural defects in gene-targeted mouse models. Figure 1, Figure 2, Figure 3, Figure 4 and Figure 5 contain high-resolution images improved from previous publications and intended for illustrative purposes of discussions made in the main body of the text. These illustrations serve as spatial and temporal references for evaluating chronological events of amelogenesis. Details about tissue processing and the preparation of fixed samples for focused ion beam scanning electron microscopy (FIB-SEM; see abbreviations) have been published elsewhere [1]. A significant limitation of this review is that we were unable to describe all published observations of enamel defects or deduce cellular mechanisms from gene-targeted mouse models. However, we created a table to highlight the genes crucial for enamel formation, and categorized the resulting defects when these genes were altered. Table 1 provides a concise summary of the primary effect of gene mutations on enamel formation, classifying these adverse effects into the following six categories: enamel agenesis, severe enamel hypoplasia (defined as a reduction of more than 50% in enamel thickness), enamel hypomineralization, dysplastic enamel organ epithelium, dysfunctional enamel organ epithelium, and minor enamel defects.

### 1.2. Ameloblast Lifecycle

Ameloblasts have a uniquely complex lifecycle. They accomplish a number of developmental processes that are extraordinary from an epithelial cell biology standpoint. Most notably, they reverse their internal cell polarity so that they can control the development of an enamel layer at their embryological bases separating them from adjacent mesenchyme by a skin-type basement membrane [3,4,5]. The skin-type basement membrane, or basal lamina (BL), is a specialized layer of the extracellular matrix situated between the epidermis and dermis. This layer plays a crucial role as an interface, enabling communication and adhesion between these two tissue types. Although all basement membranes contain common structural elements, the skin-type basement membrane has distinctive functions, which include fostering molecular interactions, facilitating tissue-layer adhesion, and supporting hair follicle regeneration.

Many improvements have been made in our understanding of the various matrix proteins and extracellular-acting proteases these cells synthesize and secrete [6,7] as well as the consequences to enamel development if these proteins and many others are dysfunctional, creating conditions of amelogenesis imperfecta [8,9,10,11,12,13,14]. Additionally, over the past few decades there has been a growing list of specific signaling, transcriptional, transport, metabolic, structural, and other proteins that elicit enamel defects if they are not expressed at the appropriate time or quantity during amelogenesis by one or more of the cells forming the enamel organ (EO) [6,15,16,17,18].

Limited progress has been made, however, in understanding the more dynamic 2- and 3-dimensional aspects of ameloblast function, including how differentiating ameloblasts become spatially organized into distinct row arrangements that are later reflected in 3D tooth- and species-specific enamel rod patterns [19,20,21,22,23]. It is equally unclear what events occur within the differentiating ameloblasts to stimulate them to remove the BM at a specific time along the interface with adjacent predentin-producing odontoblasts (Figure 1 and Figure 2), a physical act of epithelial “invasion” normally forbidden for epithelial cells to carry out [23,24,25]. There has been little progress made in understanding the role that ameloblasts may have in determining the spatial location where enamel crystallites are induced to form and in assisting the lengthening of these crystallites as the rod and interrod (IR) territories form through gradual appositional growth in the thickness of the enamel layer [14,26] (Figure 3). It is also poorly understood what internal events cause ameloblasts to form and later remove their Tomes processes (TPs) and then undergo rapid postsecretory transition (PST), leading to the death of some ameloblasts, shrinkage in cell size, and the reorganization of cytoplasmic organelles in those that survive transition [6,27] (Figure 4). It is equally unclear what factors direct these transformed ameloblasts to secrete a unique sugar-rich basal lamina on the newly formed enamel surface [28,29] (Figure 4), and then to initiate a series of repetitive modulation cycles at their distal surfaces in contact with the basal lamina involving the formation of highly invaginated and tightly sealed membrane areas between “ruffle-ended ameloblasts” that are periodically released rapidly, causing the tight seal to loosen for short intervals (“smooth-ended ameloblasts”), then the seals and invaginations reform again as ruffle-ended for the next cycle [6,27].

These modulation cycles in rodent incisors happen several times a day and last until the enamel layer becomes fully mineralized. Ameloblast modulations are needed to help promote the final volumetric expansion of mineral crystallites initially seeded in the appositional growth phase and involve processes such as ion transport for mineral growth and pH control to offset acid released as new layers of hydroxyapatite mineral form [6]. This is when the KLK4 protease is secreted from modulating ameloblasts so that the enamel matrix can be degraded into small pieces for their removal from the hardening enamel [6,7,27]. Although the focus of this discussion is on ameloblasts and events happening along their distal surfaces, it should not be forgotten that these cells form part of a stratified epithelium initially comprising four phenotypically distinct cell layers all interconnected by desmosomes that collectively function to promote amelogenesis [30]. Certain genetic dysfunctions originating within the cell layer most intimately associated with the functional base of ameloblasts—the stratum intermedium (SI)—can lead to enamel defects on their own [31,32,33].

## 2. Assessing the Mutational Effect: Direct or Indirect Impact and Phenotypic Variations 

There is, currently, a significant amount of literature demonstrating the advantages of using mouse teeth to monitor the consequences to formation of various mineralized tissues arising from altering the functional state of specific genes [34,35]. Relative to dentin and enamel formation, the molars are best suited for characterizing alterations in the rates of attrition/abrasion of teeth from long-term functional use within the oral cavity. The incisors, with their rigid linear and progressive time forward arrangement of cells comprising all stages in the dentinogenesis and amelogenesis sequences along the length of a single tooth, are ideal for determining when a developmental problem first arises and what happens to odontoblasts, enamel organ (EO) cells, and the enamel layer up to the point where a renewing segment of this tooth reaches maturity and enters the oral cavity to become a replacement cohort at the incisal edge (Figure 1).

One of the problems associated with understanding past literature on what happens within EO cells and forming an enamel layer following the loss of function of a specific gene is the assumption that all aspects of cell/matrix changes obtained—for example, from developing molars—apply equally to incisors and vice versa. This is a potentially misleading assumption regarding amelogenesis for a number of reasons [36,37,38,39]. First, it is indisputable that IDE cells undergo the exact same sequence of changes in becoming an ameloblast capable of forming and helping to mature the enamel layer, irrespective of whether they reside within an EO located on a 1st, 2nd, or 3rd molar or on one of the incisors situated in the maxilla or mandible. What are different between these teeth are the factors related to the 3D shapes and surface contours of each individual tooth and the manner in which IDE cells polarize to form group associations of secretory-stage ameloblasts that are the keystones to the eventual 3D paths ameloblasts move along in forming the complex enamel rod patterns characteristic for each tooth [21]. Secretory-stage ameloblasts do not function as individual isolated cells free to move randomly but instead function as inter-coordinated blocks of cells from which the characteristic rows of rods are created in alternating decussating patterns on rodent incisors (Figure 5) and less rigid decussating linear and diazone/parazone twisting patterns more typical of human molar teeth [40,41]. Incisor and molar enamel in mice have the same gross organization as initial, inner, outer, and final layers, but even within the same mouse total enamel thickness and the amount formed as inner enamel and as outer enamel differ on maxillary and mandibular mouse incisors [40]. In addition, maxillary incisors are more curved and heavily stained with yellow–orange iron pigment than mandibular incisors [40,42]. One of the most frequent simple and reliable indicators of a problem with amelogenesis in genetically altered mice is a loss of pigmentation and a chalky white and blunted appearance of incisal edges on the erupted portions of incisors, especially mandibular incisors [43]. Mouse mandibular incisors in general seem more sensitive to ER stress and genetic manipulations, and they often show much more extreme and disastrous effects on amelogenesis compared with maxillary incisors or molars [38,39,44,45]. Another incisor–molar difference relates to the group of cells positioned between the SI and the outer dental epithelium (ODE) called stellate reticulum (SR). As the name implies, these epithelial cells are star-shaped, with arms extending outward to form well-developed desmosomal attachments with neighboring SR cells and at the periphery with SI cells on the ameloblast side as well as with the ODE on the outermost surface of the EO where loose connective tissue and blood vessels are located [30]. There are numerous large intercellular spaces between SR cells filled with tissue fluids and rich in proteoglycans locally secreted by the SR cells [46,47,48]. The internal volume of EO occupied by SR cells and the size of intercellular spaces in molars are very large at the bell stage of tooth formation, but it is very small, just one or two cell layers thick, at an equivalent developmental time in the renewing incisors (Figure 1-1). There is a long-standing theory that the thick SR layer in molars acts as a collapsible buffer to provide space into which the tall columnar secretory-stage ameloblasts and their closely associated single layer of SI cells can relocate as they move away from the dentinoenamel junction (DEJ) as the enamel layer is being created by appositional growth [46]. This is not possible on the incisors where the SR layer is very thin and the distance from the SI cells to the outer surface of the EO is only about 30 µm. Enamel thickness on a 7-week-old mandibular mouse incisor is around 120 µm, suggesting that between the beginning and the end of the secretory stage, cohorts of EO cells (Am+SI+ SR+ODE) must reposition themselves in a coordinated fashion a distance of at least +90 µm away from their original position at the DEJ without disrupting the continuity of the whole EO (Figure 1). How this is achieved has yet to be explained.

## 3. Response of Ameloblasts to Loss of Function Mutations

### 3.1. Basic Factors Affecting Gene Alterations

It is well established that the genetic background of mice greatly influences the characteristics and severity of the altered phenotype observed following gene manipulations [43,49]. Many researchers use the N or J substrains of C57BL/6 mice for a final back crossover of several generations to stabilize the phenotype. This allows for more direct and definitive comparisons to be made between mice housing different altered genes because the animals all share the same background. Some of the earlier, and even current, research on amelogenesis has not always taken into account this issue of the genetic background of the mice being used, making interpretations or comparisons of observed phenotypes sometimes difficult to assess. Research has shown that differences in the genomic backgrounds of mouse strains complicate the interpretation of observed structural defects [50]. Furthermore, a complete gene knockout and the introduction of a premature stop codon to the same gene, resulting in a truncated mutant protein, create different biological contexts and likely have distinct functional impacts.

Another closely related problem is the embryonic and early postnatal lethal consequences arising from the loss of function of genes when they are in a homozygous null state (KO) as well as in some cases when they are in a state of conditional KO (CKO) that can occur with a *Cre* line driven, for example, by the keratin14 promotor to restrict a loss of function to skin-type epithelial cells [51,52]. Embryos and mouse pups that have no survival potential embryologically at the day of birth or within a few days or weeks after birth are clearly under various degrees of systemic stress [6,53,54]. This complicates trying to assess if a developmental problem with amelogenesis exclusively arises from the loss of function of the single lethal gene or if some of the effects observed are due to the poor and declining health of the mouse pups. These complications are being minimized by the use of ameloblast-stage-specific *Cre* mice [26,55]. 

### 3.2. Basement Membrane and Basal Lamina Essential to Enamel Formation

The literature that has accumulated over the past 50+ years has unequivocally demonstrated that the BM separating EO cells from their neighboring condensed mesenchyme is the focal point for the eventual development of the tooth shape and the spatial location where cusp tips and incisal edges will be located [56,57]. This is also the future boundary along which pre-odontoblasts will polarize and form dentin in an inward direction, and IDE cells will reverse their polarity and differentiate into ameloblasts to form the enamel layer in an outward direction. What has always seemed peculiar in the amelogenesis sequence is the need for preameloblasts to remove the BM as a preliminary step to induce initial mineralization in the predentin, followed a short time later by the start of the appositional growth of an enamel layer by fully differentiated ameloblasts, especially considering that enamel can form on bone [58]. Destruction of the BM along what will become the future DEJ is an ancient event that goes as far back as reptiles [59,60]. An even older process tracing back to fish [61] is the secretion by ameloblasts of two enamel-specific proteins, ameloblastin and enamelin, that are needed to induce and assist in the development of thin mineral ribbons characteristic of what is called the initial aprismatic layer on top of the newly mineralized dentin in mammals [1,62] (Figure 3). Once the full thickness of the enamel layer has been created, the ameloblast adds to the surface a thin replacement basal lamina rich in sugars and LAMC2 but deficient in laminin 5 and COL4A1 [28,29,63] (Figure 4 and Figure 5). Modulating maturation stage ameloblasts adhere tightly to this lamina by hemidesmosomes, and this basal lamina continues on to become the attachment site for the surface layer of squamous cells of the junctional epithelium [27,64].

### 3.3. Key Indicators of Changes in Ameloblast Polarity

The polarization of undifferentiated mesenchymal precursor cells into fully functional odontoblasts and of undifferentiated IDE epithelial cells into ameloblasts are complex processes incompletely defined or understood at present [65,66]. Two intracellular organelles that are good visual indicators of the acquisition of polarity as these cells grow in size, especially in height, are the Golgi apparatus and the often overlooked and poorly investigated centrioles and primary cilia present in both cell types [67,68,69]. These organelles remain spatially close to one another and relocate themselves to be positioned between the nucleus and the distal end of cells facing the BM [70]. Unlike the Golgi apparatus, the paired centrioles and the axoneme of primary cilia are rarely seen in random thin sections by transmission electron microscopy (TEM) within odontoblasts or ameloblasts. They are best visualized by high-resolution immunofluorescence localizations, which have revealed that the axoneme of cilia in odontoblasts point toward the pulp but they are generally directed toward the ODE in ameloblasts [71]. The extent to which cilia participate in signaling events within odontoblasts and ameloblasts has yet to be fully defined, as does any role cilia might play in other events such as Ca^2+^ signaling and in the various directional movements ameloblasts undergo as they create the enamel rods [72,73]. 

### 3.4. Ameloblasts Maintaining Cell Contact While Moving Spatially 

It is well documented that the development and maintenance of polarity by ameloblasts is strongly influenced by factors and proteins related to the forming and controlling of cell–cell and cell–matrix adhesion [65] (Table 1). The latter includes adhesion by IDE cells to the BM and to neighboring cells of the SI during the presecretory stage, a modest adhesion to the surface of the forming enamel layer by ameloblasts [66,74,75], continued strong adhesion to SI cells during the secretory stage [31], and a very strong adhesion to the sugar-rich basal lamina present on the surface of maturing enamel by modulating ameloblasts during the maturation stage [27]. The mix of proteins that are involved in making possible the adhesion between the IDE and the BM, the ameloblasts, and the forming enamel layer, the modulating ameloblasts, and the basal lamina are all different [28,29,66,75,76,77]. The genes for most of these proteins are all equally deadly to a developing embryo if any one of them is silenced [51]. The literature on tooth development in mutant forms of these proteins or in CKO mouse models that bypass the embryonic lethality problem is very spotty and hard to interpret. The one thing that is clear is that the appearance of any kind of weakness or open space between the differentiating or functional ameloblasts and the enamel surface is detrimental to amelogenesis and usually leads to dysplasia of the EO cells, including the development of various blister-like swellings/cysts, some of which resemble what is seen in glandular odontogenic cysts [1,31,62,63,78,79,80,81,82,83] (Table 1).

The final type of cell–matrix adhesion that has had variable and very conflicting descriptions in the literature is the hemidesmosome. There is wide agreement that these attachments, appearing as darkened membrane plaques [84], are present at the distal ends of modulating ameloblasts, and these hold the cells firmly against the basal lamina covering the maturing enamel surface [27]. Hemidesmosomes would be counterproductive, however, for the ameloblast cell movements required to create the 3D shape of the enamel rods and, therefore, are not seen during the secretory stage [74,77]. Hemidesmosomes are widely described to be present at the distal ends of IDE cells as they differentiate into ameloblasts. This view is mostly based on results from immunohistochemical localizations of antibodies against proteins such as plectin (HD-1) and dystonin (BP230), which form the inner membrane plaque in association with keratins 5 and/or 14 [85,86,87]. However, the membrane densities characteristic of hemidesmosomes are not seen by TEM or high-resolution FIB-SEM imaging (e.g., Figure 2) [77,85]. This suggests that although antigens for the plaque proteins may be present, they are likely to be present in amounts low enough to provide some weak adhesion to extracellular matrix proteins such as integrin a6/b4 and laminin 332 but still flexible enough to allow IDE cells to physically micro-arrange themselves into rows as they polarize into tall secretory cells capable of forming enamel rods [19,88,89].

Cell–cell adhesions within the EO include the four basic types (desmosomes, tight junctions, gap junctions, and adherens junctions) and the less well-defined focal adhesions involved in events like cell signaling or providing local attachment points for actin filaments at specific sites along the plasma membrane [90,91,92]. By far, the best and most widely described intercellular attachments for differentiating IDE cells and fully functional ameloblasts are the tight junctions that develop first at the proximal (toward the SI) then rapidly at the distal (toward the BM) ends of IDE cells as they undergo reverse polarization and dramatically increase in height to become ameloblasts [66] (Figure 1). The distal tight junctions of ameloblasts are the most highly developed, especially along the sides of ameloblasts forming boundaries between the sequential rows of cells [66]. Later in the secretory stage, the distal tight junctions become more uniform around the periphery of ameloblasts when they form the outer layer of enamel [66] (Figure 4 and Figure 5). Distal and proximal tight junctions continue on through postsecretory transition and then form a dynamic and changing association with modulating ameloblasts so that the distal tight junctions are more impermeable in ameloblasts when they are in the ruffle-ended phase and more leaky or absent when the ameloblasts are in a smooth-ended phase [27,93].

The development and distribution of desmosomes and gap junctions within the EO are less precisely defined in part because of conflicting TEM and immunohistochemical descriptions in the literature, especially relative to the presecretory and secretory stages. Desmosomes are a main structural feature easily identifiable within the SR layer between SR and ODE cells and SR and SI cells, starting early in the presecretory stage [32,85,86]. Desmosomes are then seen between SI cells and adjacent polarizing IDE cells about the time odontoblasts start making predentin. Desmosomal connections between IDE cells are more variable, as are gap junctions, as differentiation proceeds and ameloblasts start forming the enamel layer [94,95]. As noted elsewhere, a key feature of enamel formation is the group movements that ameloblasts make in creating the enamel rods. Desmosomal and gap junctions are potentially inhibitory to the alternating row movements needed to create the decussating rod pattern characteristic of rodent incisors [96]. It is for these reasons that we suspect that the desmosomal and gap junctions reported in past literature within the secretory stage were likely intercellularly arranged across the length axis of each row to allow proper signaling and movement for the group of ameloblasts moving in a direction opposite to the row of cells in front and behind it. Desmosomes are consistently seen throughout the EO during the maturation stage [32]. They are likely randomly arranged around the basolateral sides of modulating ameloblasts firmly held to the basal lamina by hemidesmosomes.

The status of adherens junctions and focal adhesions [97] within the EO are poorly defined in part because they do not form structures in ameloblasts that are visible by TEM, as are membrane plaques for hemidesmosomes and desmosomes, filamentous lines for tight junctions, and variable-shaped double-membrane inclusions/protrusions for gap junctions. Also, immunohistochemical localizations for constituent proteins have been mostly diffuse rather than focalized to membranes, as seen for tight junctions (linear staining) and gap junctions (punctate). For the most part, all cells of the EO show diffuse and variable, and sometimes linear, membrane immunostaining for proteins associated with adherens junctions and focal adhesions [86,92,98,99].

Lastly, numerous transgenic studies in mice have shown that the overexpression of certain genes, including *Ambn* and *Mmp20*, can be just as damaging to amelogenesis as too little or no expression of the same protein [100,101]. Furthermore, a complete gene knockout and the introduction of a premature stop codon to the same gene, producing a truncated mutant protein, can create different biological contexts and likely have distinct functional impacts. In some transgenic and natural mutations, the mutant protein that is formed acts as a poison or is damaging to the function of the cell that produced it.

## 4. Conclusions

Considering (a) the large number of genes participating in amelogenesis, (b) the complicated 3D movements that ameloblasts undergo in creating the enamel rods, and (c) the numerous sequential morphological changes ameloblasts and other cells of the EO undergo as they help form and mature the enamel layer, it is somewhat surprising that so few genetic problems occur during enamel development as are actually detected in vivo.

Enamel researchers have made reasonable progress in adapting genetic advances to study mouse amelogenesis using a variety of approaches that have long been a mainstay of research in other areas of hard and soft tissues. There have been major limitations, however, in technical approaches that can be used to study key events in amelogenesis because these occur while developing teeth are buried within the jawbones, limiting their accessibility to chemical and/or physical manipulations. Tooth buds and isolated enamel organs cannot be cultured in vitro for very long before the ameloblasts start to depolarlize [102]. Ameloblasts also do not tolerate being separated from other ameloblasts or from the neighboring SI cells with which they function as a unit in vivo. Putative ameloblasts cultured in vitro did not recapitulate their essential morphology and function in vivo [102]. Developing viable and stage-specific ameloblast cell lines and/or organoids are among the key challenges that, if solved, will result in greater insight into how ameloblasts make enamel and enhance the potential to engineer enamel in vitro. 

This review aimed to enhance the understanding of the challenges of using mouse models to characterize genetic mutations affecting amelogenesis. The scientific premise is that the effects of genetic mutations observed in humans may differ in mouse models. Additionally, the literature has demonstrated several key points: distinguishable structural differences exist between mouse molars and continuously growing incisors; variations in mouse genomic backgrounds influence mutational outcomes; systemic disturbances can have secondary effects on amelogenesis; the use of *Cre* lines to minimize systemic effects; and ameloblast cells must maintain polarity and cell contacts to function properly. Well-designed and thoroughly characterized gene-targeted mouse models with enamel defects are valuable tools, which will likely serve as stable platforms for intervention and therapeutic studies. Understanding the potential limitations of these tools is an important prerequisite for their effective use.

## Figures and Tables

**Table 1:** Enamel malformations associated with dysfunctional mouse genes ^1,2^.

A.	No Enamel Forms
*Ambn* KO	*Gja1* Mut
*Ash2l* CKO (Krt14)	*Nog* CKO (Krt14)
*Bmpr1a* CKO (Krt5-rtTa)	*RhoA* CKO (Krt14)
*Chip2/Bcl11b locus* KO	*Rock* CKO (Krt14)
*Eda* CKO (KRT14)	*Smo* CKO (Krt14)
*Enam* KO	*Sp6* KO
*Fst* CKO (Krt14)	*Sp7* KO
*Gdnf* KO	*Wnt3* CKO (Krt14)
**B.**	**Severe Hypoplasia (−50% or less normal thickness) Often with Poor-Quality Mineralized Material Covering Dentin**
*Acp4* Mut	*Kmt2b* CKO (Krt14)
*Acvr1* CKO (Sp7)	*Ltbp3* KO
*Amelx* KO+KI+Mut	*Mmp20* Mut
*Ctnnb1* CKO (Krt5-rtTa)	*Msx2* KO
*Ctnnd1* CKO (Krt14)	*Pitx2* CKO (Krt14-Hmgn2)
*Dlx3* CKO (Krt14)	*Postn* KO [incisors only]
*Enam* Mut	*Satb1* KO
*Evc1* KO	*Slc13a5* KO+Mut
*Fam20a* KO+CKO (Krt14)	*Stim1* CKO (Amelx)
*Fam20c* KO+CKO (Sox2)	*Tbx1* KO
**C.**	**Enamel Thickness Near Normal but with Mineralization Problems Primarily Associated with the Maturation Stage**
*Adam10* CKO (Amelx)	*Lamc2* CKO (Krt14-Dox
*Atg7* CKO (Krt14)	*Lpar6* KO
*Bcar1* CKO (Krt14)	*Mast4* KO
*Bmp2*+*Bmp4* CKO (Shh)	*Memo1* CKO (Krt14)(Pit2)
*Cftr* KO	*Nectin1* KO
*Cnnm4* KO	*Odaph* KO+Mut
*Ctnnb1* (CKI (Amelx)	*Rogdi* KO
*Gja1* CKO (Dmp1)	*Runx2* CKO (Krt14)
*Irf6* CKO (Pitx2)	*Slc4a2* KO [rods abnormal]
*Itgb1* CKO (Krt14)	*Stim1* CKO (Krt14)
*Kdf1* Mut	*Trpm7* CKO (Krt14)
*Klk4* KO	*Wdr72* KO
**D.**	**EO Cells Depolarize and Become Dysplastic, Often Having Cysts With/Without Ectopic Mineralization**
*Acp4* Mut	*Lama3* KO
*Acvr1* CKO (Sp7)	*Lamc2* CKO (Krt14-Dox)
*Ambn* KO	*Lpar6* KO
*Amelx* Mut	*Mmp20* Mut
*Bcar1* CKO (Krt14)	*Msx2* KO
*Bmp2+Bmp4* CKO (Shh)	*Nectin1* KO
*Cdc42* CKO (Krt14)	*Odaph* KO+Mut
*Enam* KO+Mut	*Postn* KO [incisors only]
*Fam20a* KO+CKO (Krt14)	*RhoA* CKO (Krt14)
*Fam20c* KO+CKO (Sox2)	*Rock1* or *2* CKO (Krt14)
*Gja1* Mut	*Slc13a5* KO+Mut
*Itgb1* CKO (Krt14)	*Wdr72* KO
*Itgb6* KO	
**E.**	**Phenotype of EO Cell Changes**
*Bmpr1a* CKO (Krt5-rtTa): EO switches to making cementum rather than enamel	
*Bsg* KO: removal of BM is delayed in presecretory stage	
*Fam20b* CKO (Krt14): EO induces the formation of supernumerary incisors	
*Fst* CKO (Krt14): EO takes on phenotype typical of Hertwig’s epithelial root sheath	
*Isl1* CKO (Krt14): enamel forms on the lingual side of incisors	
*Med1* CKO (Krt14): EO switches to making hair	
*Msx2* KO: EO starts to form keratin internally	
*Smad4* CKO (Ors2): enamel forms on top of bone rather than dentin	
*Smo* CKO (Krt14): EO becomes flattened and is squamous in appearance	
*Sox21* KO: EO starts to form keratin internally	
*Sp6* CKO (Krt5): enamel forms on lingual side of incisors	
**F.**	**Loss of Gene Function Causes Only Minor Effects on Amelogenesis**
*Adgrf2* KO	*Kdf1* Mut
*Adgrf4* KO	*Lama2* KO
*Aire* KO	*Lamc2* mut
*Amtn* KO	*Odam* KO
*Ascl5* KO	*Orai1* CKO (Krt14)
*Atg3* CKO (Krt14)	*Orai2* KO
*Atg7* CKO (Krt14)	*Phex* Mut
*Cd63* KO	*Pitx2* CKO (Krt14-Dicer1)
*Cdh2* CKO (Krt14)	*Rac1* CKO (Krt14)
*Cldn3* KO	*Relt* KO
*Cldn16* KO	*Slc10a7* KO
*Col7a1* KO [rod paths affected]	*Slc13a5* CKO (Bglap)
*Col17a1* KO [rod paths affected]	*Slc20a2* KO
*Dspp* KO+Mut	*Slc26a1/Slc26a7* KO (double)
*Fam83h* KO	*Smad3* KO
*Fgf9* KO	*Sod1* KO
*Gpr68* KO	*Sp7* CKO (Col1a1)
*Ift88* CKO (Krt14)	*Trpm7* Mut

^1^ gene name, alteration (conditional allele). ^2^ references cited in Supplementary Materials

## Supplementary Materials

The following supporting information can be downloaded at: https://www.mdpi.com/article/10.3390/ijms26104905/s1.

## Abbreviations

The following abbreviations are used in this manuscript:

3D	Three-dimensional
Am	Ameloblast(s), highly specialized tall columnar epithelial cells that form and help mature the enamel layer
BL	Basal lamina
BM	Basement membrane, which initially separates IDE cells from developing odontoblasts during the presecretory stage of amelogenesis
CKI	Conditional knockin mouse model (no expression of mutant protein until the conditional allele is activated)
CKO	Conditional knockout mouse model (no expression of native protein until the conditional allele is removed)
DEJ	Dentinoenamel junction, which starts as a basement membrane separating epithelium from mesenchyme, then becomes the union between enamel and dentin
EO	Enamel organ, a stratified epithelium initially comprising IDE/Am+SI+SR+ODE cells and Am and papillary layer cells (PL) during the maturation stage (Am+PL)
FEL	Final enamel layer
FIB-SEM	Focused ion beam scanning electron microscopy, a technique for obtaining very high-resolution imaging of object surfaces
IDE	Inner dental epithelium, a layer of low columnar epithelial cells that differentiate into Am
IEL	Initial enamel layer
IR	Interrod enamel
KO	Knockout mouse model with both genes silenced [homozygous null]
Mat	Maturation stage of amelogenesis
Mut	Gene mutation
ODE	Outer dental epithelium, a layer of polygonal-shaped epithelial cells at the surface of the EO
PST	Postsecretory transition stage of amelogenesis
SEC	Secretory stage of amelogenesis
SI	Stratum intermedium, a layer of cuboidal epithelial cells attached to the proximal ends of IDE then AM; the layer appears early in the presecretory stage and is lost as a distinct layer during postsecretory transition into the maturation stage
SR	Stellate reticulum, a variably thick layer of star-shaped epithelial cells positioned between SI and ODE
TEM	Transmission electron microscopy, a high-resolution imaging technique usually carried out on ultrathin sections cut from a sample with a diamond knife
