# Supplementary material for: Challenges of Studying Amelogenesis in Gene-Targeted Mouse Models
Source: Int J Mol Sci. 2025 May 20;26(10):4905. doi: 10.3390/ijms26104905 (PMC12112697; doi:10.3390/ijms26104905)
Supplement: Supplementary file 1 [file ijms-26-04905-s001.zip › ijms-3628575-supplementary.pdf]

Supplementary Materials

Challenges of Studying Amelogenesis in Gene-Targeted Mouse Models

Charles E. Smith[1,2], John D. Bartlett[3], James P. Simmer[2] and Jan C-C. Hu[2*]

Copyright: © 2025 by the authors. Submitted for possible open access publication under the terms and conditions of the Creative Commons Attribution (CC BY) license (https://creativecommons.org/licenses/by/4.0/).

[1]Department of Anatomy & Cell Biology, Faculty of Medicine & Health Sciences, McGill University, 3640 University St., Montreal, QC H3A 0C7, Canada

[2]Department of Biologic and Materials Sciences, School of Dentistry, University of Michigan School of Dentistry, 1011 North University Ave., Ann Arbor, MI 48190, USA

[3]Division of Biosciences, College of Dentistry, Ohio State University, 305 W. 12th Ave., Columbus, OH 43210, USA

*Correspondence: janhu@umich.edu; Tel.: +1-734-763-6769

Supplemental Table S1. Enamel Malformations Associated with Dysfunctional Mouse Genes

(gene name, alteration (conditional allele))

A. No Enamel Forms

Ambn KO	[1, 2]	*Gja1* Mut	[3, 4]
Ash2l CKO (Krt14)	[5]	*Nog* CKO (Krt14)	[6]
Bmpr1a CKO (Krt5-rtTa)	[7]	*RhoA* CKO (Krt14)	[8]
Chip2/Bcl11b locus KO	[9]	*Rock* CKO (Krt14)	[8]
Eda CKO (KRT14)	[10]	*Smo* CKO (Krt14)	[11]
Enam KO	[12, 13]	*Sp6* KO	[14]
Fst CKO (Krt14)	[10]	S*p7* KO	[15]
Gdnf KO	[16]	*Wnt3* CKO (Krt14)	[17]

B. Severe Hypoplasia (-50% or less normal thickness) Often with Poor Quality Mineralized Material Covering Dentin

Acp4 Mut	[18]	*Kmt2b* CKO (Krt14)	[19]
Acvr1 CKO (Sp7)	[20]	*Ltbp3* KO	[21]
Amelx KO+KI+Mut	[22-26]	*Mmp20* Mut	[27, 28]
Ctnnb1 CKO (Krt5-rtTa)	[29]	*Msx2* KO	[30, 31]
Ctnnd1 CKO (Krt14)	[32]	*Pitx2* CKO (Krt14-Hmgn2)	[33]
Dlx3 CKO (Krt14)	[34]	*Postn* KO [incisors only]	[35]
Enam Mut	[36]	*Satb1* KO	[37]
Evc1 KO	[38]	*Slc4a4* KO	[39]
Fam20a KO+CKO (Krt14)	[40, 41]	*Slc13a5* KO+Mut	[42, 43]
Fam20c KO+CKO (Sox2)	[40, 44]	*Stim1* CKO (Amelx)	[45]
		Tbx1 KO	[46]

C. Enamel Thickness Near Normal but Mineralization Problem Associated Primarily with the Maturation Stage

Adam10 CKO (Amelx)	[47]	*Lamc2* CKO (Krt14-Dox)	[48]
Atg7 CKO (Krt14)	[49]	*Lpar6* KO	[50]
Bcar1 CKO (Krt14)	[51]	*Mast4* KO	[52]
Bmp2+Bmp4 CKO (Shh)	[53]	*Memo1* CKO (Krt14)(Pit2)	[54]
Cftr KO	[55]	*Nectin1* KO	[56]
Cnnm4 KO	[57]	*Odaph* KO+Mut	[58, 59]
Ctnnb1 (CKI (Amelx)	[60]	*Rogdi* KO	[61]
Gja1 CKO (Dmp1)	[62]	*Runx2* CKO (Krt14)	[63]
Irf6 CKO (Pitx2)	[64]	*Slc4a2* KO [rods abnormal]	[65]
Itgb1 CKO (Krt14)	[66, 67]	*Stim1* CKO (Krt14)	[68, 69]
Kdf1 Mut	[70]	*Trpm7* CKO (Krt14)	[71]
Klk4 KO	[72]	*Wdr72* KO	[73, 74]

D. EO Cells Depolarize and Become Dysplastic Often Having Cysts With/without Ectopic Mineralization

Acp4 Mut	[18]	*Lama3* KO	[75]
Acvr1 CKO (Sp7)	[20]	*Lamc2* CKO (Krt14-Dox)	[48]
Ambn KO	[1, 2]	*Lpar6* KO	[50]
Amelx Mut	[23, 24]	*Mmp20* Mut	[27, 28]
Bcar1 CKO (Krt14)	[51]	*Msx2* KO	[30, 31]
Bmp2+Bmp4 CKO (Shh)	[53]	*Nectin1* KO	[56]
Cdc42 CKO (Krt14)	[76, 77]	*Odaph* KO+Mut	[58, 59]
Enam KO+Mut	[12, 13, 36]	*Postn* KO [incisors only]	[35]
Fam20a KO+CKO (Krt14)	[40, 41]	*RhoA* CKO (Krt14)	[8]
Fam20c KO+CKO (Sox2)	[40, 44]	*Rock1* or *2* CKO (Krt14)	[8]
Gja1 Mut	[3, 4]	*Slc13a5* KO+Mut	[42, 43]
Itgb1 CKO (Krt14)	[66, 67]	*Wdr72* KO	[73, 74]
Itgb6 KO	[78]		

E. Phenotype of EO Cells Changes

Bmpr1a CKO (Krt5-rtTa), EO switches to making cementum rather than enamel [7]

Bsg KO, removal of BM is delayed in presecretory stage [79]

Fam20b CKO (Krt14), EO induces formation of supernumerary incisors [40]

Fst CKO (Krt14), EO takes on phenotype typical of Hertwig's epithelial root sheath [10]

Isl1 CKO (Krt14), enamel forms on lingual side of incisors [80]

Med1 CKO (Krt14), EO switches to making hair [81, 82]

Msx2 KO, EO starts to form keratin internally [31]

Smad4 CKO (Ors2), enamel forms on top of bone rather than dentin [83]

Smo CKO (Krt14), EO becomes flattened squamous in appearance [11]

Sox21 KO, EO starts to form keratin internally [84]

Sp6 CKO (Krt5), enamel forms on lingual side of incisors [85]

F. Loss of Gene Function Causes Only Minor Effects on Amelogenesis

Adgrf2 KO	[86]	*Kdf1* Mut	[70]
Adgrf4 KO	[87]	*Lama2* KO	[88]
Aire KO	[89]	*Lamc2* mut	[90]
Amtn KO	[91, 92]	*Odam* KO	[93]
Ascl5 KO	[94]	*Orai1* CKO (Krt14)	[95]
Atg3 CKO (Krt14)	[96]	*Orai2* KO	[95]
Atg7 CKO (Krt14)	[49, 96]	*Phex* Mut	[97]
Cd63 KO	[98]	*Pitx2* CKO (Krt14-Dicer1)	[99]
Cdh2 CKO (Krt14)	[100]	*Rac1* CKO (Krt14)	[101]
Cldn3 KO	[102]	*Relt* KO	[103]
Cldn16 KO	[104]	*Slc10a7* KO	[105]
Col7a1 KO [rod paths affected]	[106]	*Slc13a5* CKO (Bglap)	[107]
Col17a1 KO [rod paths affected]	[108]	*Slc20a2* KO	[109]
Dspp KO+Mut	[110]	Slc24a4 KO	[111, 112]
Fam83h KO+Mut	[113, 114]	*Slc26a1/Slc26a7* KO (double)	[115]
Fgf9 KO+Mut	[116]	*Smad3* KO	[117]
Gpr68 KO	[118]	*Sod1* KO	[119]
Ift88 CKO (Krt14)	[120]	*Sp7* CKO (Col1a1)	[121]
		Trpm7 Mut	[122]

References

1. Fukumoto, S.; Kiba, T.; Hall, B.; Iehara, N.; Nakamura, T.; Longenecker, G.; Krebsbach, P. H.; Nanci, A.; Kulkarni, A. B.; Yamada, Y., Ameloblastin is a cell adhesion molecule required for maintaining the differentiation state of ameloblasts. *J Cell Biol* **2004,** 167, (5), 973-83. https://doi.org/10.1083/jcb.200409077
2. Liang, T.; Hu, Y.; Smith, C. E.; Richardson, A. S.; Zhang, H.; Yang, J.; Lin, B.; Wang, S. K.; Kim, J. W.; Chun, Y. H.; Simmer, J. P.; Hu, J. C., AMBN mutations causing hypoplastic amelogenesis imperfecta and Ambn knockout-NLS-lacZ knockin mice exhibiting failed amelogenesis and Ambn tissue-specificity. *Mol Genet Genomic Med* **2019,** 7, (9), e929. https://doi.org/10.1002/mgg3.929
3. Flenniken, A. M.; Osborne, L. R.; Anderson, N.; Ciliberti, N.; Fleming, C.; Gittens, J. E.; Gong, X. Q.; Kelsey, L. B.; Lounsbury, C.; Moreno, L.; Nieman, B. J.; Peterson, K.; Qu, D.; Roscoe, W.; Shao, Q.; Tong, D.; Veitch, G. I.; Voronina, I.; Vukobradovic, I.; Wood, G. A.; Zhu, Y.; Zirngibl, R. A.; Aubin, J. E.; Bai, D.; Bruneau, B. G.; Grynpas, M.; Henderson, J. E.; Henkelman, R. M.; McKerlie, C.; Sled, J. G.; Stanford, W. L.; Laird, D. W.; Kidder, G. M.; Adamson, S. L.; Rossant, J., A Gja1 missense mutation in a mouse model of oculodentodigital dysplasia. *Development* **2005,** 132, (19), 4375-86. https://doi.org/10.1242/dev.02011
4. Toth, K.; Shao, Q.; Lorentz, R.; Laird, D. W., Decreased levels of Cx43 gap junctions result in ameloblast dysregulation and enamel hypoplasia in Gja1Jrt/+ mice. *J Cell Physiol* **2010,** 223, (3), 601-9. https://doi.org/10.1002/jcp.22046
5. Zhu, X.; Ma, Z.; Xie, F.; Wang, J., ASH2L, Core Subunit of H3K4 Methylation Complex, Regulates Amelogenesis. *Journal of Dental Research* **2023,** 103, (1), 81-90. https://doi.org/10.1177/00220345231207309
6. Plikus, M. V.; Zeichner-David, M.; Mayer, J.-A.; Reyna, J.; Bringas, P.; Thewissen, J. G. M.; Snead, M. L.; Chai, Y.; Chuong, C.-M., Morphoregulation of teeth: modulating the number, size, shape and differentiation by tuning Bmp activity. *Evolution & Development* **2005,** 7, (5), 440-457. https://doi.org/10.1111/j.1525-142X.2005.05048.x
7. Yang, Z.; Bo, H.; Lizheng, Q.; Xinyu, T.; Lei, S.; Yanqiu, Z.; Lindsey, W.; Ying, L.; Q., F. J.; Fong, C. J. Y.; Fen, W.; and Liu, F., Cessation of Epithelial Bmp Signaling Switches the Differentiation of Crown Epithelia to the Root Lineage in a β-Catenin-Dependent Manner. *Molecular and Cellular Biology* **2013,** 33, (23), 4732-4744. https://doi.org/10.1128/MCB.00456-13
8. Otsu, K.; Ida-Yonemochi, H.; Fujiwara, N.; Harada, H., The Semaphorin 4D-RhoA-Akt Signal Cascade Regulates Enamel Matrix Secretion in Coordination With Cell Polarization During Ameloblast Differentiation. *J Bone Miner Res* **2016,** 31, (11), 1943-1954. https://doi.org/10.1002/jbmr.2876
9. Golonzhka, O.; Metzger, D.; Bornert, J.-M.; Bay, B. K.; Gross, M. K.; Kioussi, C.; Leid, M., Ctip2/Bcl11b controls ameloblast formation during mammalian odontogenesis. *Proceedings of the National Academy of Sciences* **2009,** 106, (11), 4278-4283. https://doi.org/doi:10.1073/pnas.0900568106
10. Wang, X. P.; Suomalainen, M.; Jorgez, C. J.; Matzuk, M. M.; Werner, S.; Thesleff, I., Follistatin regulates enamel patterning in mouse incisors by asymmetrically

inhibiting BMP signaling and ameloblast differentiation. *Dev Cell* **2004,** 7, (5), 719-30. https://doi.org/10.1016/j.devcel.2004.09.012
11. Gritli-Linde, A.; Bei, M.; Maas, R.; Zhang, X. M.; Linde, A.; McMahon, A. P., Shh signaling within the dental epithelium is necessary for cell proliferation, growth and polarization. *Development* **2002,** 129, (23), 5323-37. https://doi.org/10.1242/dev.00100
12. Hu, J. C.; Hu, Y.; Smith, C. E.; McKee, M. D.; Wright, J. T.; Yamakoshi, Y.; Papagerakis, P.; Hunter, G. K.; Feng, J. Q.; Yamakoshi, F.; Simmer, J. P., Enamel defects and ameloblast-specific expression in Enam knock-out/lacz knock-in mice. *J Biol Chem* **2008,** 283, (16), 10858-71. https://doi.org/10.1074/jbc.M710565200
13. Hu, J. C.; Hu, Y.; Lu, Y.; Smith, C. E.; Lertlam, R.; Wright, J. T.; Suggs, C.; McKee, M. D.; Beniash, E.; Kabir, M. E.; Simmer, J. P., Enamelin is critical for ameloblast integrity and enamel ultrastructure formation. *PLoS One* **2014,** 9, (3), e89303. https://doi.org/10.1371/journal.pone.0089303
14. Nakamura, T.; de Vega, S.; Fukumoto, S.; Jimenez, L.; Unda, F.; Yamada, Y., Transcription factor epiprofin is essential for tooth morphogenesis by regulating epithelial cell fate and tooth number. *J Biol Chem* **2008,** 283, (8), 4825-33. https://doi.org/10.1074/jbc.M708388200
15. Bae, J. M.; Clarke, J. C.; Rashid, H.; Adhami, M. D.; McCullough, K.; Scott, J. S.; Chen, H.; Sinha, K. M.; de Crombrugghe, B.; Javed, A., Specificity Protein 7 Is Required for Proliferation and Differentiation of Ameloblasts and Odontoblasts. *J Bone Miner Res* **2018,** 33, (6), 1126-1140. https://doi.org/10.1002/jbmr.3401
16. de Vicente, J. C.; Cabo, R.; Ciriaco, E.; Laurà, R.; Naves, F. J.; Silos-Santiago, I.; Vega, J. A., Impaired dental cytodifferentiation in glial cell-line derived growth factor (GDNF) deficient mice. *Ann Anat* **2002,** 184, (1), 85-92. https://doi.org/10.1016/s0940-9602(02)80041-3
17. Millar, S. E.; Koyama, E.; Reddy, S. T.; Andl, T.; Gaddapara, T.; Piddington, R.; Gibson, C. W., Over- and ectopic expression of Wnt3 causes progressive loss of ameloblasts in postnatal mouse incisor teeth. *Connect Tissue Res* **2003,** 44 Suppl 1, 124-9.
18. Liang, T.; Wang, S.-K.; Smith, C.; Zhang, H.; Hu, Y.; Seymen, F.; Koruyucu, M.; Kasimoglu, Y.; Kim, J.-W.; Zhang, C.; Saunders, T. L.; Simmer, J. P.; Hu, J. C. C., Enamel defects in Acp4R110C/R110C mice and human ACP4 mutations. *Scientific Reports* **2022,** 12, (1), 16477. https://doi.org/10.1038/s41598-022-20684-9
19. Lee, J.-M.; Jung, H.; Tang, Q.; An, W.; Lee, S.-K.; Lee, J. W.; Park, Y.; Kwon, H.-J. E., Mll4 regulates tooth enamel development. *bioRxiv* **2024**, 2024.08.20.608898. https://doi.org/10.1101/2024.08.20.608898
20. Zhang, X.; Shi, C.; Zhao, H.; Zhou, Y.; Hu, Y.; Yan, G.; Liu, C.; Li, D.; Hao, X.; Mishina, Y.; Liu, Q.; Sun, H., Distinctive role of ACVR1 in dentin formation: requirement for dentin thickness in molars and prevention of osteodentin formation in incisors of mice. *Journal of Molecular Histology* **2019,** 50, (1), 43-61. https://doi.org/10.1007/s10735-018-9806-z
21. Huckert, M.; Stoetzel, C.; Morkmued, S.; Laugel-Haushalter, V.; Geoffroy, V.; Muller, J.; Clauss, F.; Prasad, M. K.; Obry, F.; Raymond, J. L.; Switala, M.; Alembik, Y.; Soskin, S.; Mathieu, E.; Hemmerlé, J.; Weickert, J. L.; Dabovic, B. B.; Rifkin, D. B.; Dheedene, A.; Boudin, E.; Caluseriu, O.; Cholette, M. C.; McLeod, R.; Antequera, R.; Gellé, M. P.; Coeuriot, J. L.; Jacquelin, L. F.; Bailleul-Forestier, I.; Manière, M. C.; Van Hul, W.;

Bertola, D.; Dollé, P.; Verloes, A.; Mortier, G.; Dollfus, H.; Bloch-Zupan, A., Mutations in the latent TGF-beta binding protein 3 (LTBP3) gene cause brachyolmia with amelogenesis imperfecta. *Hum Mol Genet* **2015,** 24, (11), 3038-49. https://doi.org/10.1093/hmg/ddv053
22. Gibson, C. W.; Yuan, Z. A.; Hall, B.; Longenecker, G.; Chen, E.; Thyagarajan, T.; Sreenath, T.; Wright, J. T.; Decker, S.; Piddington, R.; Harrison, G.; Kulkarni, A. B., Amelogenin-deficient mice display an amelogenesis imperfecta phenotype. *J Biol Chem* **2001,** 276, (34), 31871-5. https://doi.org/10.1074/jbc.M104624200
23. Barron, M. J.; Brookes, S. J.; Kirkham, J.; Shore, R. C.; Hunt, C.; Mironov, A.; Kingswell, N. J.; Maycock, J.; Shuttleworth, C. A.; Dixon, M. J., A mutation in the mouse Amelx tri-tyrosyl domain results in impaired secretion of amelogenin and phenocopies human X-linked amelogenesis imperfecta. *Hum Mol Genet* **2010,** 19, (7), 1230-47. https://doi.org/10.1093/hmg/ddq001
24. Brookes, S. J.; Barron, M. J.; Boot-Handford, R.; Kirkham, J.; Dixon, M. J., Endoplasmic reticulum stress in amelogenesis imperfecta and phenotypic rescue using 4-phenylbutyrate. *Hum Mol Genet* **2014,** 23, (9), 2468-80. https://doi.org/10.1093/hmg/ddt642
25. Hu, Y.; Smith, C. E.; Cai, Z.; Donnelly, L. A.-J.; Yang, J.; Hu, J. C.-C.; Simmer, J. P., Enamel ribbons, surface nodules, and octacalcium phosphate in C57BL/6 Amelx mice and Amelx lyonization. *Molecular Genetics & Genomic Medicine* **2016,** 4, (6), 641-661. https://doi.org/10.1002/mgg3.252
26. Smith, C. E.; Hu, Y.; Hu, J. C.; Simmer, J. P., Ultrastructure of early amelogenesis in wild-type, Amelx(-/-), and Enam(-/-) mice: enamel ribbon initiation on dentin mineral and ribbon orientation by ameloblasts. *Mol Genet Genomic Med* **2016,** 4, (6), 662-683. https://doi.org/10.1002/mgg3.253
27. Bartlett, J. D.; Smith, C. E., Modulation of cell-cell junctional complexes by matrix metalloproteinases. *J Dent Res* **2013,** 92, (1), 10-7. https://doi.org/10.1177/0022034512463397
28. Bartlett, J. D.; Smith, C. E.; Hu, Y.; Ikeda, A.; Strauss, M.; Liang, T.; Hsu, Y. H.; Trout, A. H.; McComb, D. W.; Freeman, R. C.; Simmer, J. P.; Hu, J. C., MMP20-generated amelogenin cleavage products prevent formation of fan-shaped enamel malformations. *Sci Rep* **2021,** 11, (1), 10570. https://doi.org/10.1038/s41598-021-90005-z
29. Guan, X.; Xu, M.; Millar, S. E.; Bartlett, J. D., Beta-catenin is essential for ameloblast movement during enamel development. *Eur J Oral Sci* **2016,** 124, (3), 221-7. https://doi.org/10.1111/eos.12261
30. Satokata, I.; Ma, L.; Ohshima, H.; Bei, M.; Woo, I.; Nishizawa, K.; Maeda, T.; Takano, Y.; Uchiyama, M.; Heaney, S.; Peters, H.; Tang, Z.; Maxson, R.; Maas, R., Msx2 deficiency in mice causes pleiotropic defects in bone growth and ectodermal organ formation. *Nat Genet* **2000,** 24, (4), 391-5. https://doi.org/10.1038/74231
31. Nakatomi, M.; Ida-Yonemochi, H.; Nakatomi, C.; Saito, K.; Kenmotsu, S.; Maas, R. L.; Ohshima, H., Msx2 Prevents Stratified Squamous Epithelium Formation in the Enamel Organ. *J Dent Res* **2018,** 97, (12), 1355-1364. https://doi.org/10.1177/0022034518777746
32. Bartlett, J. D.; Dobeck, J. M.; Tye, C. E.; Perez-Moreno, M.; Stokes, N.; Reynolds, A. B.; Fuchs, E.; Skobe, Z., Targeted p120-catenin ablation disrupts dental enamel

development. *PLoS One* **2010,** 5:12703, (9). https://doi.org/10.1371/journal.pone.0012703
33. Li, X.; Venugopalan, S. R.; Cao, H.; Pinho, F. O.; Paine, M. L.; Snead, M. L.; Semina, E. V.; Amendt, B. A., A model for the molecular underpinnings of tooth defects in Axenfeld-Rieger syndrome. *Hum Mol Genet* **2014,** 23, (1), 194-208. https://doi.org/10.1093/hmg/ddt411
34. Duverger, O.; Ohara, T.; Bible, P. W.; Zah, A.; Morasso, M. I., DLX3-Dependent Regulation of Ion Transporters and Carbonic Anhydrases is Crucial for Enamel Mineralization. *J Bone Miner Res* **2017,** 32, (3), 641-653. https://doi.org/10.1002/jbmr.3022
35. Rios, H.; Koushik, S. V.; Wang, H.; Wang, J.; Zhou, H.-M.; Lindsley, A.; Rogers, R.; Chen, Z.; Maeda, M.; Kruzynska-Frejtag, A.; Feng, J. Q.; Conway, S. J., periostin Null Mice Exhibit Dwarfism, Incisor Enamel Defects, and an Early-Onset Periodontal Disease-Like Phenotype. *Molecular and Cellular Biology* **2005,** 25, (24), 11131-11144. https://doi.org/10.1128/MCB.25.24.11131-11144.2005
36. Brookes, S. J.; Barron, M. J.; Smith, C. E. L.; Poulter, J. A.; Mighell, A. J.; Inglehearn, C. F.; Brown, C. J.; Rodd, H.; Kirkham, J.; Dixon, M. J., Amelogenesis imperfecta caused by N-terminal enamelin point mutations in mice and men is driven by endoplasmic reticulum stress. *Hum Mol Genet* **2017,** 26, (10), 1863-1876. https://doi.org/10.1093/hmg/ddx090
37. Zhang, Y.; Zheng, L.; Le, M.; Nakano, Y.; Chan, B.; Huang, Y.; Torbaty, P. M.; Kohwi, Y.; Marcucio, R.; Habelitz, S.; Den Besten, P. K.; Kohwi-Shigematsu, T., SATB1 establishes ameloblast cell polarity and regulates directional amelogenin secretion for enamel formation. *BMC Biology* **2019,** 17, (1), 104. https://doi.org/10.1186/s12915-019-0722-9
38. Ruiz-Perez, V. L.; Blair, H. J.; Rodriguez-Andres, M. E.; Blanco, M. J.; Wilson, A.; Liu, Y. N.; Miles, C.; Peters, H.; Goodship, J. A., Evc is a positive mediator of Ihh-regulated bone growth that localises at the base of chondrocyte cilia. *Development* **2007,** 134, (16), 2903-12. https://doi.org/10.1242/dev.007542
39. Lacruz, R. S.; Nanci, A.; White, S. N.; Wen, X.; Wang, H.; Zalzal, S. F.; Luong, V. Q.; Schuetter, V. L.; Conti, P. S.; Kurtz, I.; Paine, M. L., The Sodium Bicarbonate Cotransporter (NBCe1) Is Essential for Normal Development of Mouse Dentition *. *Journal of Biological Chemistry* **2010,** 285, (32), 24432-24438. https://doi.org/10.1074/jbc.M110.115188
40. Vogel, P.; Hansen, G. M.; Read, R. W.; Vance, R. B.; Thiel, M.; Liu, J.; Wronski, T. J.; Smith, D. D.; Jeter-Jones, S.; Brommage, R., Amelogenesis imperfecta and other biomineralization defects in Fam20a and Fam20c null mice. *Vet Pathol* **2012,** 49, (6), 998-1017. https://doi.org/10.1177/0300985812453177
41. Li, L. L.; Liu, P. H.; Xie, X. H.; Ma, S.; Liu, C.; Chen, L.; Qin, C. L., Loss of epithelial FAM20A in mice causes amelogenesis imperfecta, tooth eruption delay and gingival overgrowth. *Int J Oral Sci* **2016,** 8, (2), 98-109. https://doi.org/10.1038/ijos.2016.14
42. Irizarry, A. R.; Yan, G.; Zeng, Q.; Lucchesi, J.; Hamang, M. J.; Ma, Y. L.; Rong, J. X., Defective enamel and bone development in sodium-dependent citrate transporter (NaCT) Slc13a5 deficient mice. *PLoS One* **2017,** 12, (4), e0175465. https://doi.org/10.1371/journal.pone.0175465

43. Simmer, J. P.; Hu, J. C.; Hu, Y.; Zhang, S.; Liang, T.; Wang, S. K.; Kim, J. W.; Yamakoshi, Y.; Chun, Y. H.; Bartlett, J. D.; Smith, C. E., A genetic model for the secretory stage of dental enamel formation. *J Struct Biol* **2021,** 213, (4), 107805. https://doi.org/10.1016/j.jsb.2021.107805
44. Liu, J.; Saiyin, W.; Xie, X.; Mao, L.; Li, L., Ablation of Fam20c causes amelogenesis imperfecta via inhibiting Smad dependent BMP signaling pathway. *Biol Direct* **2020,** 15, (1), 16. https://doi.org/10.1186/s13062-020-00270-7
45. Said, R.; Mortazavi, H.; Cooper, D.; Ovens, K.; McQuillan, I.; Papagerakis, S.; Papagerakis, P., Deciphering the functions of Stromal Interaction Molecule-1 in amelogenesis using AmelX-iCre mice. *Frontiers in Physiology* **2023,** 14:1100714. https://doi.org/10.3389/fphys.2023.1100714
46. Catón, J.; Luder, H. U.; Zoupa, M.; Bradman, M.; Bluteau, G.; Tucker, A. S.; Klein, O.; Mitsiadis, T. A., Enamel-free teeth: Tbx1 deletion affects amelogenesis in rodent incisors. *Dev Biol* **2009,** 328, (2), 493-505. https://doi.org/10.1016/j.ydbio.2009.02.014
47. Shahid, S.; Hu, Y.; Mohamed, F.; Rizzotto, L.; Layana, M. C.; Fleming, D. T.; Papagerakis, P.; Foster, B. L.; Simmer, J. P.; Bartlett, J. D., ADAM10 Expression by Ameloblasts Is Essential for Proper Enamel Formation. *International Journal of Molecular Sciences* **2024,** 25, (23), 13184. https://doi.org/10.3390/ijms252313184
48. Wazen, R. M.; Viegas-Costa, L. C.; Fouillen, A.; Moffatt, P.; Adair-Kirk, T. L.; Senior, R. M.; Nanci, A., Laminin γ2 knockout mice rescued with the human protein exhibit enamel maturation defects. *Matrix Biology* **2016,** 52-54, 207-218. https://doi.org/10.1016/j.matbio.2016.03.002
49. Sukseree, S.; Yacine, S. U.; Reinhard, G.; Florian, G.; Maria, Q. D. R.; D., M. J.; D., B. J.; Erwin, T.; and Eckhart, L., ATG7 is essential for secretion of iron from ameloblasts and normal growth of murine incisors during aging. *Autophagy* **2020,** 16, (10), 1851-1857. https://doi.org/10.1080/15548627.2019.1709764
50. Inaba, A.; Harada, H.; Ikezaki, S.; Kumakami-Sakano, M.; Arai, H.; Azumane, M.; Ohshima, H.; Morikawa, K.; Kano, K.; Aoki, J.; Otsu, K., LPA6-RhoA signals regulate junctional complexes for polarity and morphology establishment of maturation stage ameloblasts. *Journal of Oral Biosciences* **2022,** 64, (1), 85-92. https://doi.org/10.1016/j.job.2022.01.004
51. Inoue, A.; Kiyoshima, T.; Yoshizaki, K.; Nakatomi, C.; Nakatomi, M.; Ohshima, H.; Shin, M.; Gao, J.; Tsuru, K.; Okabe, K.; Nakamura, I.; Honda, H.; Matsuda, M.; Takahashi, I.; Jimi, E., Deletion of epithelial cell-specific p130Cas impairs the maturation stage of amelogenesis. *Bone* **2022,** 154, 116210. https://doi.org/10.1016/j.bone.2021.116210
52. Lee, D.-J.; Kim, P.; Kim, H.-Y.; Park, J.; Lee, S.-J.; An, H.; Heo, J. S.; Lee, M.-J.; Ohshima, H.; Mizuno, S.; Takahashi, S.; Jung, H.-S.; Kim, S.-J., MAST4 regulates stem cell maintenance with DLX3 for epithelial development and amelogenesis. *Experimental & Molecular Medicine* **2024,** 56, (7), 1606-1619. https://doi.org/10.1038/s12276-024-01264-5
53. Reibring, C.-G.; El Shahawy, M.; Hallberg, K.; Harfe, B. D.; Linde, A.; Gritli-Linde, A., Loss of BMP2 and BMP4 Signaling in the Dental Epithelium Causes Defective Enamel Maturation and Aberrant Development of Ameloblasts. *International Journal of Molecular Sciences* **2022,** 23, (11), 6095. https://doi.org/10.3390/ijms23116095

54. Kiel, M.; Wuebker, S.; Remy, M. T.; Riemondy, K. A.; Smith, F.; Carey, C. M.; Williams, T.; Van Otterloo, E., MEMO1 Is Required for Ameloblast Maturation and Functional Enamel Formation. *J Dent Res* **2023,** 102, (11), 1261-1271. https://doi.org/10.1177/00220345231185758
55. Wright, J. T.; Kiefer, C. L.; Hall, K. I.; Grubb, B. R., Abnormal enamel development in a cystic fibrosis transgenic mouse model. *J Dent Res* **1996,** 75, (4), 966-73. https://doi.org/10.1177/00220345960750041101
56. Barron, M. J.; Brookes, S. J.; Draper, C. E.; Garrod, D.; Kirkham, J.; Shore, R. C.; Dixon, M. J., The cell adhesion molecule nectin-1 is critical for normal enamel formation in mice. *Hum Mol Genet* **2008,** 17, (22), 3509-20. https://doi.org/10.1093/hmg/ddn243
57. Yamazaki, D.; Funato, Y.; Miura, J.; Sato, S.; Toyosawa, S.; Furutani, K.; Kurachi, Y.; Omori, Y.; Furukawa, T.; Tsuda, T.; Kuwabata, S.; Mizukami, S.; Kikuchi, K.; Miki, H., Basolateral Mg2+ extrusion via CNNM4 mediates transcellular Mg2+ transport across epithelia: a mouse model. *PLoS Genet* **2013,** 9, (12), e1003983. https://doi.org/10.1371/journal.pgen.1003983
58. Ji, Y.; Li, C.; Tian, Y.; Gao, Y.; Dong, Z.; Xiang, L.; Xu, Z.; Gao, Y.; Zhang, L., Maturation stage enamel defects in Odontogenesis-associated phosphoprotein (Odaph) deficient mice. *Developmental Dynamics* **2021,** 250, (10), 1505-1517. https://doi.org/10.1002/dvdy.336
59. Liang, T.; Hu, Y.; Kawasaki, K.; Zhang, H.; Zhang, C.; Saunders, T. L.; Simmer, J. P.; Hu, J. C., Odontogenesis-associated phosphoprotein truncation blocks ameloblast transition into maturation in Odaph(C41*/C41*) mice. *Sci Rep* **2021,** 11, (1), 1132. https://doi.org/10.1038/s41598-020-80912-y
60. Fan, L.; Deng, S.; Sui, X.; Liu, M.; Cheng, S.; Wang, Y.; Gao, Y.; Chu, C. H.; Zhang, Q., Constitutive activation of β-catenin in ameloblasts leads to incisor enamel hypomineralization. *J Mol Histol* **2018,** 49, (5), 499-507. https://doi.org/10.1007/s10735-018-9788-x
61. Jimenez-Armijo, A.; Morkmued, S.; Ahumada, J. T.; Kharouf, N.; de Feraudy, Y.; Gogl, G.; Riet, F.; Niederreither, K.; Laporte, J.; Birling, M. C.; Selloum, M.; Herault, Y.; Hernandez, M.; Bloch-Zupan, A., The Rogdi knockout mouse is a model for Kohlschütter-Tönz syndrome. *Sci Rep* **2024,** 14, (1), 445. https://doi.org/10.1038/s41598-023-50870-2
62. Al-Ansari, S.; Jalali, R.; Plotkin, L. I.; Bronckers, A.; DenBesten, P.; Zhang, Y.; Raber-Durlacher, J. E.; de Lange, J.; Rozema, F. R., The Importance of Connexin 43 in Enamel Development and Mineralization. *Front Physiol* **2018,** 9, 750. https://doi.org/10.3389/fphys.2018.00750
63. Chu, Q.; Gao, Y.; Gao, X.; Dong, Z.; Song, W.; Xu, Z.; Xiang, L.; Wang, Y.; Zhang, L.; Li, M.; Gao, Y., Ablation of Runx2 in Ameloblasts Suppresses Enamel Maturation in Tooth Development. *Scientific Reports* **2018,** 8, (1), 9594. https://doi.org/10.1038/s41598-018-27873-5
64. Chu, E. Y.; Tamasas, B.; Fong, H.; Foster, B. L.; LaCourse, M. R.; Tran, A. B.; Martin, J. F.; Schutte, B. C.; Somerman, M. J.; Cox, T. C., Full Spectrum of Postnatal Tooth Phenotypes in a Novel Irf6 Cleft Lip Model. *J Dent Res* **2016,** 95, (11), 1265-73. https://doi.org/10.1177/0022034516656787
65. Lyaruu, D. M.; Bronckers, A. L.; Mulder, L.; Mardones, P.; Medina, J. F.; Kellokumpu, S.; Oude Elferink, R. P.; Everts, V., The anion exchanger Ae2 is required for enamel

maturation in mouse teeth. *Matrix Biol* **2008,** 27, (2), 119-27. https://doi.org/10.1016/j.matbio.2007.09.006

66. Chen, B.; Goodman, E.; Lu, Z.; Bandyopadhyay, A.; Magraw, C.; He, T.; Raghavan, S., Function of beta1 integrin in oral epithelia and tooth bud morphogenesis. *J Dent Res* **2009,** 88, (6), 539-44. https://doi.org/10.1177/0022034509338008
67. Saito, K.; Fukumoto, E.; Yamada, A.; Yuasa, K.; Yoshizaki, K.; Iwamoto, T.; Saito, M.; Nakamura, T.; Fukumoto, S., Interaction between fibronectin and β1 integrin is essential for tooth development. *PLoS One* **2015,** 10, (4), e0121667. https://doi.org/10.1371/journal.pone.0121667
68. Eckstein, M.; Vaeth, M.; Fornai, C.; Vinu, M.; Bromage, T. G.; Nurbaeva, M. K.; Sorge, J. L.; Coelho, P. G.; Idaghdour, Y.; Feske, S.; Lacruz, R. S., Store-operated Ca(2+) entry controls ameloblast cell function and enamel development. *JCI Insight* **2017,** 2, (6), e91166. https://doi.org/10.1172/jci.insight.91166
69. Furukawa, Y.; Haruyama, N.; Nikaido, M.; Nakanishi, M.; Ryu, N.; Oh-Hora, M.; Kuremoto, K.; Yoshizaki, K.; Takano, Y.; Takahashi, I., Stim1 Regulates Enamel Mineralization and Ameloblast Modulation. *J Dent Res* **2017,** 96, (12), 1422-1429. https://doi.org/10.1177/0022034517719872
70. Li, P.; Zeng, B.; Xie, W.; Xiao, X.; Lin, L.; Yu, D.; Zhao, W., Enamel Structure Defects in Kdf1 Missense Mutation Knock-in Mice. *Biomedicines* **2023,** 11, (2). https://doi.org/10.3390/biomedicines11020482
71. Shin, M.; Matsushima, A.; Kajiya, H.; Okamoto, F.; Ogata, K.; Oka, K.; Ohshima, H.; Bartlett, J. D.; Okabe, K., Conditional knockout of transient receptor potential melastatin 7 in the enamel epithelium: Effects on enamel formation. *Eur J Oral Sci* **2023,** 131, (2), e12920. https://doi.org/10.1111/eos.12920
72. Simmer, J. P.; Hu, Y.; Lertlam, R.; Yamakoshi, Y.; Hu, J. C., Hypomaturation enamel defects in Klk4 knockout/LacZ knockin mice. *J Biol Chem* **2009,** 284, (28), 19110-21. https://doi.org/10.1074/jbc.M109.013623
73. Wang, S. K.; Hu, Y.; Yang, J.; Smith, C. E.; Nunez, S. M.; Richardson, A. S.; Pal, S.; Samann, A. C.; Hu, J. C.; Simmer, J. P., Critical roles for WDR72 in calcium transport and matrix protein removal during enamel maturation. *Mol Genet Genomic Med* **2015,** 3, (4), 302-19. https://doi.org/10.1002/mgg3.143
74. Katsura, K.; Nakano, Y.; Zhang, Y.; Shemirani, R.; Li, W.; Den Besten, P., WDR72 regulates vesicle trafficking in ameloblasts. *Scientific Reports* **2022,** 12, (1), 2820. https://doi.org/10.1038/s41598-022-06751-1
75. Ryan, M. C.; Lee, K.; Miyashita, Y.; Carter, W. G., Targeted disruption of the LAMA3 gene in mice reveals abnormalities in survival and late stage differentiation of epithelial cells. *J Cell Biol* **1999,** 145, (6), 1309-23. https://doi.org/10.1083/jcb.145.6.1309
76. Zheng, J.; Nie, X.; He, L.; Yoon, A. J.; Wu, L.; Zhang, X.; Vats, M.; Schiff, M. D.; Xiang, L.; Tian, Z.; Ling, J.; Mao, J. J., Epithelial Cdc42 Deletion Induced Enamel Organ Defects and Cystogenesis. *J Dent Res* **2018,** 97, (12), 1346-1354. https://doi.org/10.1177/0022034518779546
77. Zhang, J.; Gao, J.; Zeng, X.; Wang, Z.; Chen, C.; Rong, C.; Li, S.; Cai, L.; Wang, L.; Zhang, L.; Tian, Z., A novel Cdc42-YAP-fibronectin signaling axis regulates ameloblast differentiation during early enamel formation. *Biochimica et Biophysica*

Acta (BBA) - Molecular Basis of Disease **2025,** 1871, (2), 167570. https://doi.org/10.1016/j.bbadis.2024.167570
78. Mohazab, L.; Koivisto, L.; Jiang, G.; Kytömäki, L.; Haapasalo, M.; Owen, G. R.; Wiebe, C.; Xie, Y.; Heikinheimo, K.; Yoshida, T.; Smith, C. E.; Heino, J.; Häkkinen, L.; McKee, M. D.; Larjava, H., Critical role for αvβ6 integrin in enamel biomineralization. *J Cell Sci* **2013,** 126, (Pt 3), 732-44. https://doi.org/10.1242/jcs.112599
79. Khaddam, M.; Huet, E.; Vallée, B.; Bensidhoum, M.; Le Denmat, D.; Filatova, A.; Jimenez-Rojo, L.; Ribes, S.; Lorenz, G.; Morawietz, M.; Rochefort, G. Y.; Kiesow, A.; Mitsiadis, T. A.; Poliard, A.; Petzold, M.; Gabison, E. E.; Menashi, S.; Chaussain, C., EMMPRIN/CD147 deficiency disturbs ameloblast–odontoblast cross-talk and delays enamel mineralization. *Bone* **2014,** 66, 256-266. https://doi.org/10.1016/j.bone.2014.06.019
80. Naveau, A.; Zhang, B.; Meng, B.; Sutherland, M. T.; Prochazkova, M.; Wen, T.; Marangoni, P.; Jones, K. B.; Cox, T. C.; Ganss, B.; Jheon, A. H.; Klein, O. D., Isl1 Controls Patterning and Mineralization of Enamel in the Continuously Renewing Mouse Incisor. *J Bone Miner Res* **2017,** 32, (11), 2219-2231. https://doi.org/10.1002/jbmr.3202
81. Yoshizaki, K.; Hu, L.; Nguyen, T.; Sakai, K.; He, B.; Fong, C.; Yamada, Y.; Bikle, D. D.; Oda, Y., Ablation of coactivator Med1 switches the cell fate of dental epithelia to that generating hair. *PLoS One* **2014,** 9, (6), e99991. https://doi.org/10.1371/journal.pone.0099991
82. Yoshizaki, K.; Hu, L.; Nguyen, T.; Sakai, K.; Ishikawa, M.; Takahashi, I.; Fukumoto, S.; DenBesten, P. K.; Bikle, D. D.; Oda, Y.; Yamada, Y., Mediator 1 contributes to enamel mineralization as a coactivator for Notch1 signaling and stimulates transcription of the alkaline phosphatase gene. *J Biol Chem* **2017,** 292, (33), 13531-13540. https://doi.org/10.1074/jbc.M117.780866
83. Li, J.; Huang, X.; Xu, X.; Mayo, J.; Bringas, P., Jr.; Jiang, R.; Wang, S.; Chai, Y., SMAD4-mediated WNT signaling controls the fate of cranial neural crest cells during tooth morphogenesis. *Development* **2011,** 138, (10), 1977-89. https://doi.org/10.1242/dev.061341
84. Saito, K.; Michon, F.; Yamada, A.; Inuzuka, H.; Yamaguchi, S.; Fukumoto, E.; Yoshizaki, K.; Nakamura, T.; Arakaki, M.; Chiba, Y.; Ishikawa, M.; Okano, H.; Thesleff, I.; Fukumoto, S., Sox21 Regulates Anapc10 Expression and Determines the Fate of Ectodermal Organ. *iScience* **2020,** 23, (7), 101329. https://doi.org/10.1016/j.isci.2020.101329
85. Nakamura, T.; Jimenez-Rojo, L.; Koyama, E.; Pacifici, M.; de Vega, S.; Iwamoto, M.; Fukumoto, S.; Unda, F.; Yamada, Y., Epiprofin Regulates Enamel Formation and Tooth Morphogenesis by Controlling Epithelial-Mesenchymal Interactions During Tooth Development. *J Bone Miner Res* **2017,** 32, (3), 601-610. https://doi.org/10.1002/jbmr.3024
86. Chiba, Y.; Yoshizaki, K.; Sato, H.; Ikeuchi, T.; Rhodes, C.; Chiba, M.; Saito, K.; Nakamura, T.; Iwamoto, T.; Yamada, A.; Yamada, Y.; Fukumoto, S., Deficiency of G protein-coupled receptor Gpr111/Adgrf2 causes enamel hypomineralization in mice by alteration of the expression of kallikrein-related peptidase 4 (Klk4) during pH cycling process. *The FASEB Journal* **2023,** 37, (4), e22861. https://doi.org/10.1096/fj.202202053R

87. Chiba, Y.; Yoshizaki, K.; Saito, K.; Ikeuchi, T.; Iwamoto, T.; Rhodes, C.; Nakamura, T.; de Vega, S.; Morell, R. J.; Boger, E. T.; Martin, D.; Hino, R.; Inuzuka, H.; Bleck, C. K. E.; Yamada, A.; Yamada, Y.; Fukumoto, S., G protein–coupled receptor Gpr115 (Adgrf4) is required for enamel mineralization mediated by ameloblasts. *Journal of Biological Chemistry* **2020,** 295, (45), 15328-15341. https://doi.org/10.1074/jbc.RA120.014281
88. Yuasa, K.; Fukumoto, S.; Kamasaki, Y.; Yamada, A.; Fukumoto, E.; Kanaoka, K.; Saito, K.; Harada, H.; Arikawa-Hirasawa, E.; Miyagoe-Suzuki, Y.; Takeda, S.; Okamoto, K.; Kato, Y.; Fujiwara, T., Laminin α2 Is Essential for Odontoblast Differentiation Regulating Dentin Sialoprotein Expression*. *Journal of Biological Chemistry* **2004,** 279, (11), 10286-10292. https://doi.org/10.1074/jbc.M310013200
89. Gruper, Y.; Wolff, A. S. B.; Glanz, L.; Spoutil, F.; Marthinussen, M. C.; Osickova, A.; Herzig, Y.; Goldfarb, Y.; Aranaz-Novaliches, G.; Dobeš, J.; Kadouri, N.; Ben-Nun, O.; Binyamin, A.; Lavi, B.; Givony, T.; Khalaila, R.; Gome, T.; Wald, T.; Mrazkova, B.; Sochen, C.; Besnard, M.; Ben-Dor, S.; Feldmesser, E.; Orlova, E. M.; Hegedűs, C.; Lampé, I.; Papp, T.; Felszeghy, S.; Sedlacek, R.; Davidovich, E.; Tal, N.; Shouval, D. S.; Shamir, R.; Guillonneau, C.; Szondy, Z.; Lundin, K. E. A.; Osicka, R.; Prochazka, J.; Husebye, E. S.; Abramson, J., Autoimmune amelogenesis imperfecta in patients with APS-1 and coeliac disease. *Nature* **2023,** 624, (7992), 653-662. https://doi.org/10.1038/s41586-023-06776-0
90. Bubier, J. A.; Sproule, T. J.; Alley, L. M.; Webb, C. M.; Fine, J. D.; Roopenian, D. C.; Sundberg, J. P., A mouse model of generalized non-Herlitz junctional epidermolysis bullosa. *J Invest Dermatol* **2010,** 130, (7), 1819-28. https://doi.org/10.1038/jid.2010.46
91. Nakayama, Y.; Holcroft, J.; Ganss, B., Enamel Hypomineralization and Structural Defects in Amelotin-deficient Mice. *J Dent Res* **2015,** 94, (5), 697-705. https://doi.org/10.1177/0022034514566214
92. Núñez, S. M.; Chun, Y.-H. P.; Ganss, B.; Hu, Y.; Richardson, A. S.; Schmitz, J. E.; Fajardo, R.; Yang, J.; Hu, J. C. C.; Simmer, J. P., Maturation stage enamel malformations in Amtn and Klk4 null mice. *Matrix Biology* **2016,** 52-54, 219-233. https://doi.org/10.1016/j.matbio.2015.11.007
93. Wazen, R. M.; Moffatt, P.; Ponce, K. J.; Kuroda, S.; Nishio, C.; Nanci, A., Inactivation of the Odontogenic ameloblast-associated gene affects the integrity of the junctional epithelium and gingival healing. *Eur Cell Mater* **2015,** 30, 187-99. https://doi.org/10.22203/ecm.v030a13
94. Chiba, Y.; He, B.; Yoshizaki, K.; Rhodes, C.; Ishijima, M.; Bleck, C. K. E.; Stempinski, E.; Chu, E. Y.; Nakamura, T.; Iwamoto, T.; de Vega, S.; Saito, K.; Fukumoto, S.; Yamada, Y., The transcription factor AmeloD stimulates epithelial cell motility essential for tooth morphology. *Journal of Biological Chemistry* **2019,** 294, (10), 3406-3418. https://doi.org/10.1074/jbc.RA118.005298
95. Eckstein, M.; Vaeth, M.; Aulestia, F. J.; Costiniti, V.; Kassam, S. N.; Bromage, T. G.; Pedersen, P.; Issekutz, T.; Idaghdour, Y.; Moursi, A. M.; Feske, S.; Lacruz, R. S., Differential regulation of Ca(2+) influx by ORAI channels mediates enamel mineralization. *Sci Signal* **2019,** 12, (578). https://doi.org/10.1126/scisignal.aav4663
96. Iwaya, C.; Suzuki, A.; Shim, J.; Ambrose, C. G.; Iwata, J., Autophagy Plays a Crucial Role in Ameloblast Differentiation. *Journal of Dental Research* **2023,** 102, (9), 1047-1057. https://doi.org/10.1177/00220345231169220

97. Mohamed, F. F.; Hoac, B.; Phanrungsuwan, A.; Tan, M. H.; Giovani, P. A.; Ghiba, S.; Murshed, M.; Foster, B. L.; McKee, M. D., Contributions of increased osteopontin and hypophosphatemia to dentoalveolar defects in osteomalacic Hyp mice. *Bone* **2023,** 176, 116886. https://doi.org/10.1016/j.bone.2023.116886
98. Lacruz, R. S.; Brookes, S. J.; Wen, X.; Jimenez, J. M.; Vikman, S.; Hu, P.; White, S. N.; Lyngstadaas, S. P.; Okamoto, C. T.; Smith, C. E.; Paine, M. L., Adaptor protein complex 2-mediated, clathrin-dependent endocytosis, and related gene activities, are a prominent feature during maturation stage amelogenesis. *J Bone Miner Res* **2013,** 28, (3), 672-87. https://doi.org/10.1002/jbmr.1779
99. Cao, H.; Jheon, A.; Li, X.; Sun, Z.; Wang, J.; Florez, S.; Zhang, Z.; McManus, M. T.; Klein, O. D.; Amendt, B. A., The Pitx2:miR-200c/141:noggin pathway regulates Bmp signaling and ameloblast differentiation. *Development* **2013,** 140, (16), 3348-59. https://doi.org/10.1242/dev.089193
100. Guan, X.; Bidlack, F. B.; Stokes, N.; Bartlett, J. D., E-cadherin can replace N-cadherin during secretory-stage enamel development. *PLoS One* **2014,** 9, (7), e102153. https://doi.org/10.1371/journal.pone.0102153
101. Huang, Z.; Kim, J.; Lacruz, R. S.; Bringas, P., Jr.; Glogauer, M.; Bromage, T. G.; Kaartinen, V. M.; Snead, M. L., Epithelial-specific knockout of the Rac1 gene leads to enamel defects. *Eur J Oral Sci* **2011,** 119 Suppl 1, (0 1), 168-76. https://doi.org/10.1111/j.1600-0722.2011.00904.x
102. Bardet, C.; Ribes, S.; Wu, Y.; Diallo, M. T.; Salmon, B.; Breiderhoff, T.; Houillier, P.; Müller, D.; Chaussain, C., Claudin Loss-of-Function Disrupts Tight Junctions and Impairs Amelogenesis. *Frontiers in Physiology* **2017,** Volume 8 - 2017. https://doi.org/10.3389/fphys.2017.00326
103. Kim, J. W.; Zhang, H.; Seymen, F.; Koruyucu, M.; Hu, Y.; Kang, J.; Kim, Y. J.; Ikeda, A.; Kasimoglu, Y.; Bayram, M.; Zhang, C.; Kawasaki, K.; Bartlett, J. D.; Saunders, T. L.; Simmer, J. P.; Hu, J. C., Mutations in RELT cause autosomal recessive amelogenesis imperfecta. *Clin Genet* **2019,** 95, (3), 375-383. https://doi.org/10.1111/cge.13487
104. Bardet, C.; Courson, F.; Wu, Y.; Khaddam, M.; Salmon, B.; Ribes, S.; Thumfart, J.; Yamaguti, P. M.; Rochefort, G. Y.; Figueres, M. L.; Breiderhoff, T.; Garcia-Castaño, A.; Vallée, B.; Le Denmat, D.; Baroukh, B.; Guilbert, T.; Schmitt, A.; Massé, J. M.; Bazin, D.; Lorenz, G.; Morawietz, M.; Hou, J.; Carvalho-Lobato, P.; Manzanares, M. C.; Fricain, J. C.; Talmud, D.; Demontis, R.; Neves, F.; Zenaty, D.; Berdal, A.; Kiesow, A.; Petzold, M.; Menashi, S.; Linglart, A.; Acevedo, A. C.; Vargas-Poussou, R.; Müller, D.; Houillier, P.; Chaussain, C., Claudin-16 Deficiency Impairs Tight Junction Function in Ameloblasts, Leading to Abnormal Enamel Formation. *J Bone Miner Res* **2016,** 31, (3), 498-513. https://doi.org/10.1002/jbmr.2726
105. Dubail, J.; Huber, C.; Chantepie, S.; Sonntag, S.; Tüysüz, B.; Mihci, E.; Gordon, C. T.; Steichen-Gersdorf, E.; Amiel, J.; Nur, B.; Stolte-Dijkstra, I.; van Eerde, A. M.; van Gassen, K. L.; Breugem, C. C.; Stegmann, A.; Lekszas, C.; Maroofian, R.; Karimiani, E. G.; Bruneel, A.; Seta, N.; Munnich, A.; Papy-Garcia, D.; De La Dure-Molla, M.; Cormier-Daire, V., SLC10A7 mutations cause a skeletal dysplasia with amelogenesis imperfecta mediated by GAG biosynthesis defects. *Nat Commun* **2018,** 9, (1), 3087. https://doi.org/10.1038/s41467-018-05191-8
106. Umemoto, H.; Akiyama, M.; Domon, T.; Nomura, T.; Shinkuma, S.; Ito, K.; Asaka, T.; Sawamura, D.; Uitto, J.; Uo, M.; Kitagawa, Y.; Shimizu, H., Type VII collagen

deficiency causes defective tooth enamel formation due to poor differentiation of ameloblasts. *Am J Pathol* **2012,** 181, (5), 1659-71. https://doi.org/10.1016/j.ajpath.2012.07.018
107. Dirckx, N.; Zhang, Q.; Chu, E. Y.; Tower, R. J.; Li, Z.; Guo, S.; Yuan, S.; Khare, P. A.; Zhang, C.; Verardo, A.; Alejandro, L. O.; Park, A.; Faugere, M. C.; Helfand, S. L.; Somerman, M. J.; Riddle, R. C.; de Cabo, R.; Le, A.; Schmidt-Rohr, K.; Clemens, T. L., A specialized metabolic pathway partitions citrate in hydroxyapatite to impact mineralization of bones and teeth. *Proc Natl Acad Sci U S A* **2022,** 119, (45), e2212178119. https://doi.org/10.1073/pnas.2212178119
108. Asaka, T.; Akiyama, M.; Domon, T.; Nishie, W.; Natsuga, K.; Fujita, Y.; Abe, R.; Kitagawa, Y.; Shimizu, H., Type XVII collagen is a key player in tooth enamel formation. *Am J Pathol* **2009,** 174, (1), 91-100. https://doi.org/10.2353/ajpath.2009.080573
109. Beck-Cormier, S.; Lelliott, C. J.; Logan, J. G.; Lafont, D. T.; Merametdjian, L.; Leitch, V. D.; Butterfield, N. C.; Protheroe, H. J.; Croucher, P. I.; Baldock, P. A.; Gaultier-Lintia, A.; Maugars, Y.; Nicolas, G.; Banse, C.; Normant, S.; Magne, N.; Gérardin, E.; Bon, N.; Sourice, S.; Guicheux, J.; Beck, L.; Williams, G. R.; Bassett, J. H. D., Slc20a2, Encoding the Phosphate Transporter PiT2, Is an Important Genetic Determinant of Bone Quality and Strength. *J Bone Miner Res* **2019,** 34, (6), 1101-1114. https://doi.org/10.1002/jbmr.3691
110. Verdelis, K.; Szabo-Rogers, H. L.; Xu, Y.; Chong, R.; Kang, R.; Cusack, B. J.; Jani, P.; Boskey, A. L.; Qin, C.; Beniash, E., Accelerated enamel mineralization in Dspp mutant mice. *Matrix Biol* **2016,** 52-54, 246-259. https://doi.org/10.1016/j.matbio.2016.01.003
111. Parry, D. A.; Poulter, J. A.; Logan, C. V.; Brookes, S. J.; Jafri, H.; Ferguson, C. H.; Anwari, B. M.; Rashid, Y.; Zhao, H.; Johnson, C. A.; Inglehearn, C. F.; Mighell, A. J., Identification of mutations in SLC24A4, encoding a potassium-dependent sodium/calcium exchanger, as a cause of amelogenesis imperfecta. *Am J Hum Genet* **2013,** 92, (2), 307-12. https://doi.org/10.1016/j.ajhg.2013.01.003
112. Bronckers, A. L.; Jalali, R.; Lytton, J., Reduced Protein Expression of the Na(+)/Ca(2+)+K(+)-Exchanger (SLC24A4) in Apical Plasma Membranes of Maturation Ameloblasts of Fluorotic Mice. *Calcif Tissue Int* **2017,** 100, (1), 80-86. https://doi.org/10.1007/s00223-016-0197-4
113. Wang, S. K.; Hu, Y.; Smith, C. E.; Yang, J.; Zeng, C.; Kim, J. W.; Hu, J. C.; Simmer, J. P., The Enamel Phenotype in Homozygous Fam83h Truncation Mice. *Mol Genet Genomic Med* **2019,** 7, (6), e724. https://doi.org/10.1002/mgg3.724
114. Zheng, X.; Huang, W.; He, Z.; Li, Y.; Li, S.; Song, Y., Effects of Fam83h truncation mutation on enamel developmental defects in male C57/BL6J mice. *Bone* **2023,** 166, 116595. https://doi.org/10.1016/j.bone.2022.116595
115. Yin, K.; Guo, J.; Lin, W.; Robertson, S. Y. T.; Soleimani, M.; Paine, M. L., Deletion of Slc26a1 and Slc26a7 Delays Enamel Mineralization in Mice. *Front Physiol* **2017,** 8, 307. https://doi.org/10.3389/fphys.2017.00307
116. Tang, L.; Chen, M.; Wu, M.; Liang, H.; Ge, H.; Ma, Y.; Shen, Y.; Lu, S.; Shen, C.; Zhang, H.; Zhang, C.; Wang, Z., Fgf9 promotes incisor dental epithelial stem cell survival and enamel formation. *Stem Cell Res Ther* **2024,** 15, (1), 293. https://doi.org/10.1186/s13287-024-03894-y

117. Yokozeki, M.; Afanador, E.; Nishi, M.; Kaneko, K.; Shimokawa, H.; Yokote, K.; Deng, C.; Tsuchida, K.; Sugino, H.; Moriyama, K., Smad3 is required for enamel biomineralization. *Biochemical and Biophysical Research Communications* **2003,** 305, (3), 684-690. https://doi.org/10.1016/S0006-291X(03)00806-4
118. Parry, D. A.; Smith, C. E.; El-Sayed, W.; Poulter, J. A.; Shore, R. C.; Logan, C. V.; Mogi, C.; Sato, K.; Okajima, F.; Harada, A.; Zhang, H.; Koruyucu, M.; Seymen, F.; Hu, J. C.; Simmer, J. P.; Ahmed, M.; Jafri, H.; Johnson, C. A.; Inglehearn, C. F.; Mighell, A. J., Mutations in the pH-Sensing G-protein-Coupled Receptor GPR68 Cause Amelogenesis Imperfecta. *Am J Hum Genet* **2016,** 99, (4), 984-990. https://doi.org/10.1016/j.ajhg.2016.08.020
119. Xu, Y.; Zhang, Y.; Zheng, J.; Xu, M.; Yang, Y.; Guo, W., ROS-Mediated Enamel Formation Disturbance Characterized by Alternative Cervical Loop Cell Proliferation and Downregulation of RhoA/ROCK in Ameloblasts. *Oxid Med Cell Longev* **2022,** 2022, 5769679. https://doi.org/10.1155/2022/5769679
120. Kudo, T.; Kawasaki, M.; Kawasaki, K.; Meguro, F.; Nihara, J.; Honda, I.; Kitamura, M.; Fujita, A.; Osawa, K.; Ichikawa, K.; Nagai, T.; Ishida, Y.; Sharpe, P. T.; Maeda, T.; Saito, I.; Ohazama, A., Ift88 regulates enamel formation via involving Shh signaling. *Oral Dis* **2023,** 29, (4), 1622-1631. https://doi.org/10.1111/odi.14162
121. Zhang, H.; Jiang, Y.; Qin, C.; Liu, Y.; Ho, S. P.; Feng, J. Q., Essential role of osterix for tooth root but not crown dentin formation. *J Bone Miner Res* **2015,** 30, (4), 742-6. https://doi.org/10.1002/jbmr.2391
122. Ogata, K.; Tsumuraya, T.; Oka, K.; Shin, M.; Okamoto, F.; Kajiya, H.; Katagiri, C.; Ozaki, M.; Matsushita, M.; Okabe, K., The crucial role of the TRPM7 kinase domain in the early stage of amelogenesis. *Sci Rep* **2017,** 7, (1), 18099. https://doi.org/10.1038/s41598-017-18291-0
